# Genetic Architecture of Sexual Selection: QTL Mapping of Male Song and Female Receiver Traits in an Acoustic Moth

**DOI:** 10.1371/journal.pone.0044554

**Published:** 2012-09-05

**Authors:** Denis Limousin, Réjane Streiff, Brigitte Courtois, Virginie Dupuy, Sylvain Alem, Michael D. Greenfield

**Affiliations:** 1 Centre National de la Recherche Scientifique (CNRS), UMR 7261 (IRBI), Université François Rabelais de Tours, Parc de Grandmont, Tours, France; 2 Institut National de la Recherche Agronomique (INRA), UMR CBGP (INRA – IRD – CIRAD – Montpellier SupAgro), Campus International de Baillarguet, CS 30016, Montferrier sur Lez, France; 3 Centre International de Recherches en Agronomie pour le Développement (CIRAD), UMR AGAP, Montpellier, France; The University of Queensland, St. Lucia, Australia

## Abstract

Models of indirect (genetic) benefits sexual selection predict linkage disequilibria between genes that influence male traits and female preferences, owing to non-random mate choice or physical linkage. Such linkage disequilibria can accelerate the evolution of traits and preferences to exaggerated levels. Both theory and recent empirical findings on species recognition suggest that such linkage disequilibria may result from physical linkage or pleiotropy, but very little work has addressed this possibility within the context of sexual selection. We studied the genetic architecture of sexually selected traits by analyzing signals and preferences in an acoustic moth, *Achroia grisella*, in which males attract females with a train of ultrasound pulses and females prefer loud songs and a fast pulse rhythm. Both male signal characters and female preferences are repeatable and heritable traits. Moreover, female choice is based largely on male song, while males do not appear to provide direct benefits at mating. Thus, some genetic correlation between song and preference traits is expected. We employed a standard crossing design between inbred lines and used AFLP markers to build a linkage map for this species and locate quantitative trait loci (QTL) that influence male song and female preference. Our analyses mostly revealed QTLs of moderate strength that influence various male signal and female receiver traits, but one QTL was found that exerts a major influence on the pulse-pair rate of male song, a critical trait in female attraction. However, we found no evidence of specific co-localization of QTLs influencing male signal and female receiver traits on the same linkage groups. This finding suggests that the sexual selection process would proceed at a modest rate in *A. grisella* and that evolution toward exaggerated character states may be tempered. We suggest that this equilibrium state may be more the norm than the exception among animal species.

## Introduction

Models of indirect (genetic) benefits sexual selection predict that male signal traits and female preference traits may evolve toward exaggerated levels of development when substantial additive genetic variance exists for both traits [1]. Moreover, the rate of trait evolution and the level of trait exaggeration attained are expected to be influenced by the degree of linkage disequilibrium between the traits [2]. Thus, where signals evolve by means of an indirect benefits mechanism, some amount of genetic covariance between signal and preference traits should be present [3], [4].

The linkage disequilibrium purported to occur between signal and preference traits may originate from the very process of non-random mate choice [5], or it may arise because genes that influence these two traits happen to be linked physically [6]. That is, a given gene may pleiotropically control both signal and preference, or the separate genes that control these two traits are very tightly linked and reside in a chromosomal region experiencing little recombination. Here, we note that tight physical linkage itself might not necessarily initiate genetic covariance, but it can prevent or delay its loss once present.

Whereas the functioning of indirect benefits mechanisms can be confirmed mathematically [7], [8], events that arise in natural populations may potentially interfere with actual occurrence of the process. For example, when genotype×environment interaction is present, environmental change and migration may seriously degrade any linkage disequilibrium between a signal and a preference that had previously developed owing to non-random mate choice [9], [10]. On the other hand, a linkage disequilibrium that reflects physical linkage would resist these factors [6]. Consequently, this latter origin of linkage disequilibrium between genes controlling signals and preferences could be the prevalent one, to the extent that indirect benefits mechanisms operate in nature.

To date, a number of biologists have studied the genetic architecture of signaling and preferences operating in the context of species recognition, population divergence, and speciation [11]. Recently, several of these studies have revealed evidence implicating physical linkage [12]–[14], including pleiotropy [15], between genes controlling signaling and associated preferences. These results support the notion of ‘genetic coupling’ [16], which contends that population divergence is more likely to occur when the same gene(s) that control male signals also control female preferences for those signals: Such physical linkage can facilitate the evolution of novel male signaling variants by ensuring that when these signals do appear the corresponding female preference variants will also be present. But despite this above flurry of activity at the species recognition level, very little empirical work has examined the analogous question of genetic architecture of signals and preferences operating at the finer level of evaluating potential mates among those individuals recognized as conspecific. Some of this neglect simply reflects the difficulty of systematically measuring the preference phenotypes of the large number of females that would be necessary for genetic analysis: These measurements may be relatively easy to accomplish when they pertain to species recognition, e.g. basic responses to sound frequencies in male song [17], [18], to chemical compounds in male pheromone [19], etc., which are normally subject to stabilizing selection [20]. However, female responses and preferences in the context of mate evaluation may be an order of magnitude more subtle, and measuring these traits may entail determining the shape of a ‘preference function’ (sensu [21]) or the relative emphases that a female places on the several characters of a male signal (e.g. rhythm, intensity) when making an overall assessment [22], [23]. This difficulty would be particularly acute in species using multi-modal signals, where individual components of a male display may be evaluated by females in specific ways [24]. Moreover, these evaluation protocols may change according to female condition and the environment [25].

Here, we present the findings of a study on the genetic architecture of male signals and female preferences in an acoustic pyralid moth, *Achroia grisella*, in which males broadcast an advertisement song attractive to females [26]. Previous behavioral tests and playback experiments in the laboratory have confirmed the presence of female choice and that a substantial proportion of it involves evaluation of male song characters [27], [28]. Importantly, no evidence suggests that female *A. grisella* obtain any direct benefits, e.g. spermatophore nutrients, by mating with a given male. Quantitative genetic studies conducted on several populations indicate substantial additive genetic variance (V_A_) and heritability for various male song characters [29], [30] as well as for the nature of female preference for those characters [31], [32]. These several factors imply that an indirect benefits mechanism of sexual selection probably operates in *A. grisella*.

To examine the genetic architecture of the male song and female preference for male song, we bred hybrid and backcross generations from two different inbred lines of *A. grisella*, and we measured the song and preference phenotype in these generations. We then created a linkage map developed from amplified fragment-length polymorphism (AFLP) molecular markers genotyped among the backcross generation. We applied a standard QTL mapping approach to these phenotypic and genotypic data and thereby identified quantitative trait loci responsible for the song and preference traits as well as several developmental traits that had been measured incidentally. Thus, we determined the number of loci having moderate to major influences on male signal and female preference traits, their distribution within the genome, and whether the QTLs for any male song characters and the corresponding female preference are colocalized on the same linkage group (chromosome) and in the same region within the group. To date, this study represents one of the very few attempts to investigate the genetic architecture of both male signal characters and female mate preference within the same population. As such, it examines a fundamental expectation of the mechanisms of indirect benefits sexual selection, and it offers some insight on how these mechanisms might function in natural populations.

## Materials and Methods

### Ecology and reproductive behavior of *Achroia grisella*

*A. grisella* (lesser waxmoth) are obligate symbionts of the western honeybee (*Apis mellifera*) and are found in most geographic regions of the world to which honeybees have spread [33]. The moth larvae feed on honeycomb and other organic material at honeybee colonies [33], and they normally infest colonies that have low worker populations owing to more serious parasites or other causes of decline. Mating activities occur in, on, or near the colony, and females subsequently oviposit there, provided that some food remains [34]. The adult longevity of *A. grisella* is markedly short. Female and male adults survive only 7 and 10–14 days, respectively, and they neither feed nor drink [34].

Males gather in small aggregations at bee colonies and advertise to females with a courtship song that consists of an incessant train of ultrasonic pulses [26]. They produce their song for 6–10 h each night until death while remaining stationary on the substrate and fanning their wings at 35–50 cycles per second (rate as measured at 25°C). Wing-fanning causes a pair of small tymbal structures situated at the base of each forewing to resonate, once on the upstroke of the wings and once on the downstroke. Each resonance yields a brief (80–130 µs) pulse of relatively intense sound [peak amplitude = 90–95 dB SPL (sound pressure level) at 1 cm; 0 dB SPL = 20 µPa] comprising frequencies from 70–130 kHz. Because the wings are not in perfect synchrony during both the upstrokes and downstrokes, two distinct pulses, separated by a brief ‘asynchrony interval’, are normally produced by the left and right tymbals. Thus, a wing-fanning male generates pulse pairs at 70–100 pulse pairs s^−1^, twice the rate of cycles of wing movement.

Female *A. grisella* respond to the acoustic displays of potential mates in male aggregations by running toward the acoustic source, rather than flying, and they may exhibit such phonotaxis over a distance up to 1 m [34]. Previous playback experiments in which synthetic calling song stimuli were broadcast in a laboratory arena showed that females prefer male songs whose pulses have greater peak amplitude, are delivered at a faster rate, and that include longer silent gaps (asynchrony intervals) within the pulse pairs [27], [28], [35]. Females usually mate only once and become unreceptive thereafter, whereas males can mate once per day for several consecutive days. It is unlikely that females obtain direct benefits by virtue of mating with a given male, as there is no paternal care, no observations indicate transfer of parasites at mating, the spermatophore is quite small (<0.5% of male body mass), and the rarity of remating suggests that females do not seek spermatophore material other than sperm.

Experiments conducted with several *A. grisella* populations showed that considerable variation exists among individual males in the song characters that influence attractiveness to females [27], [35] as well as in overall attractiveness of male song [28], [36]. Quantitative genetic analyses of these populations have also shown that the various male song characters and overall attractiveness are repeatable within individual males [37], and breeding experiments have confirmed their heritability (h^2^ values ranging from 0.20–0.56) and evolvability [29], [30]. A smaller amount of work with female *A. grisella* has revealed analogous phenotypic variation, repeatability, and heritability in female preference traits (H^2^ values ranging from 0.21–0.40)[31], [32]. These traits entailed the minimum (threshold) value of a song character (pulse-pair rate) that elicited female response [32] and the relative emphasis that females placed on different song characters (pulse-pair rate and asynchrony interval) when evaluating male song [31]. Our assumption that an indirect benefits mechanism of sexual selection operates in *A. grisella* is based on these above findings.

### Moth stocks and rearing

We used two inbred lines, one initially developed from a population collected in Kansas (USA) and the other in Florida (USA). Each line was derived from a full-sib crossing scheme maintained for more than 30 generations. We used these two lines because they were part of a larger study on the genetics of sexually selected traits in *A. grisella*., originated from populations separated by more than 2000 km, and had somewhat different characteristics. Florida males were 33% heavier than Kansas males (16 vs 12 mg), whereas Kansas males sang with a faster pulse-pair rate (76 vs 71 pulse pairs·s^−1^) and at a higher amplitude (sound pressure level 25% higher, linear scale). Within each line, coefficients of variation for these three parameters were approximately 12, 7 and 25%, respectively.

Both larvae and adults were kept in an environmental chamber at 26°C±1°C under a 12:12 h L:D photoperiod. The moth larvae were reared on a standard diet containing wheat, corn and rye flours, water, glycerol, nutritional yeast, honey and beeswax [38].

### Crossing scheme

Female Kansas (KS) moths were crossed to male Florida (FL) moths and the (F_1_) male hybrid progeny of this cross were paired with Kansas females to produce (F_2_) backcross individuals. Only F_1_ males were used in this design, since Lepidoptera females lack recombination and do not help to produce a distance-based recombination map [39]. Combining male and female progeny has proved to be a powerful strategy in some cases for QTL discovery, but due to experimental limitations we restricted our study to progeny issued from F_1_ males (see mapping analysis below). Backcross females and males were sampled during the pupal stage and kept individually in 30-ml plastic cups to ensure that the eclosing individuals experienced a similar social environment. This individual rearing was essential because females usually mate only once and become sexually unreceptive thereafter. A preliminary genotyping of a subset of the Kansas and Florida lines revealed that they were not as homozygous as would have been expected after 30 generations of full-sib breeding, because some AFLP bands were still present in parents of one brood and not in the other, suggesting that some heterozygosity existed. This unexpected outcome of the breeding protocol did not allow us to pool the several broods and analyze them as a single entity. We thus kept the broods with the largest sizes to produce individual maps of each brood, and we then linked the resulting genetic maps through common markers. Among 24 broods produced, we kept the two broods (Xt7 and Xt19) with the largest number of backcross individuals surviving to the adult stage, i.e. 68 individuals (35 males, 33 females) for the Xt7 brood, and 52 individuals (26 males, 26 females) for the Xt19 brood.

### Phenotype analysis

#### Measurement of developmental traits

We noted the developmental time (Dev), measured in days from oviposition to adult eclosion, and the mass (M) (±0.005 mg, determined with a Mettler Toledo AX105 DeltaRange balance) immediately following eclosion of all insects. Body mass of males and females were scored as separate parameters (M_m_ and M_f_) (see **Table 1**).

**Table 1:** Trait codes, descriptions, and units of measurement.

Trait code	Description and unit of measurement
(developmental traits)	
M	Body mass at adult eclosion, all individuals; mg
M_m_	Body mass, males; mg
M_f_	Body mass, females; mg
Dev	Duration of development from oviposition to adult eclosion; d
(male signal traits)	
PR	Pulse-pair rate; pairs·s^−1^
PA	Mean peak amplitude; arbitrary linear units
AI	Mean asynchrony interval; µs
(female receiver traits)	
Pref	Female preference for the low-PR signal; number of trials in which this choice is expressed
T	Duration of female response, measured as the interval from a female's release to her arrival at a loudspeaker; s

#### Measurement of the male signal trait

For recording song, males were kept in small screen cages (1.5 cm diameter, 2.0 cm height) placed in an acoustically insulated chamber with environmental conditions identical to those during rearing except that diffuse red light (25 W, incandescent) provided illumination. Preliminary recordings confirmed that males sang normally in these cages and that the screen did not modify the acoustic parameters of the song [37]. We placed a barrier of acoustic insulation foam between neighboring males and maintained a minimum distance of 30 cm between adjacent males to prevent a male's song from being influenced by acoustic interactions with neighbors [40]. This design also attenuated the effects of neighbors' songs (crosstalk) in the recordings of a focal male. We allowed the males to acclimate in the chamber for at least 15 min prior to recording. We used a condenser ultrasound microphone (model CM16/CMPA; Avisoft Bioacoustics; Berlin, Germany; frequency response: ±3 dB, 20–150 kHz), positioned 20 cm from the male and oriented toward him to record his song. The microphone output was digitized with an analogue: digital converter (model UltraSoundGate 416–200; Avisoft Bioacoustics) at 16 bits and 500,000 samples•s^−1^, and we saved a 30-sec sample of this digitized song to a file on a laptop computer using signal processing software (BatSound Pro 4.0; Petterson Elektronik AB; Uppsala, Sweden). From the spectrogram produced from each sampled male, we selected a 1-s segment in the middle of the recording for analysis of acoustic parameters. Our only criterion was that the 1-s segment did not include brief silent gaps that reflected missing pulse pairs in an otherwise continuous train. Earlier work on the repeatability of male song in *A. grisella* [37] confirmed that short recordings such as these were representative of an individual's signaling. We determined the repetition rate of pulse pairs (PR) for the entire segment, the duration of the asynchrony interval (AI), measured from the onset of the first pulse to the onset of the second pulse of a pair, for each pulse-pair, and the peak amplitude (PA), measured in arbitrary linear units, for each pulse. Mean values of AI and PA were then calculated for each male from his 1-s recording (**Table 1**). Earlier studies of *A. grisella* had indicated positive and negative correlations, respectively, between peak amplitude and pulse-pair rate and a male's body mass [30]. We therefore computed the least-squares linear regression of peak amplitude and of pulse-pair rate on body mass and then calculated the residual peak amplitude (PA_res_) and pulse-pair rate (PR_res_) values for each male. We included these residual values among the phenotypic parameters in our QTL analysis in order to examine song characters that were unlikely to be simple artifacts of body size.

#### Measurement of the female preference trait

We conducted all tests of female responses during the initial 6 h of the photoperiodic night, the diel interval during which mating activities in *A. grisella* are maximum. Because *A. grisella* adults neither feed nor drink and female lifespan is only 5–7 d, we tested females within 30 h of their eclosion to avoid measuring unreceptive or senescing individuals. All of our playback experiments used a choice protocol in which a female was released in the center of an 80-cm diameter screen arena and presented with simultaneous broadcasts of synthetic male song stimuli from two loudspeakers situated just outside the arena and separated by an azimuth of 120°. The central axes of the loudspeakers were level with the female in the center of the arena, and each loudspeaker was oriented directly toward her. The female was given 120 s in which to orient and arrive within a 10-cm radius of a loudspeaker. All playback tests were conducted in a second acoustically insulated chamber maintained under conditions similar to the chamber used for song recording. Females were brought to the chamber at least 30 min prior to testing, and were held in an acoustically insulated box, isolated from synthetic male song, at all times except during their playback trials. Individual females were tested six times with at least 30 min between successive trials to avoid habituation. Only data from females that responded to a song stimulus in each of the six tests were retained for analysis.

Our analysis focused on determining whether some females emphasized one signal character in their evaluation of males, while other females emphasized another. A previous study showed that females evaluate the acoustic power ( = mean amplitude×pulse-pair rate) of the male signal and generally prefer songs broadcast with greater power [41]. But when power is held constant, it may be possible to determine a female's emphasis on one or the other of the two components of power. To evaluate this aspect of female preference and to build a ‘preference index’ for each female, we created two playback stimuli that differed in pulse-pair rate (PR) and amplitude (PA) but broadcast the same acoustic power. One signal had a high PR and a low PA, while the other had a low PR and a high PA. For each of a female's 6 tests, we noted her choice of playback stimulus (high PR or high PA) and the time required for her to reach the loudspeaker. Following the 6 tests, we estimated the mean time she spent reaching her chosen loudspeaker (T, **Table 1**), and we calculated her preference index as the number of trials in which she chose the high PA signal (Pref, **Table 1**). The maximum value of this preference index was therefore 6, when a female chose the high PA signal in all 6 tests, and the minimum value was 0. We interpreted values >3 as an indication of an emphasis on amplitude in evaluation of male song and values <3 as an indication of an emphasis on pulse-pair rate in song evaluation; these cutoff values were chosen in order to retain a sufficient sample for QTL analysis.

We designed the 2 synthetic stimuli used to determine the female preference index based on the range of songs observed among males in the parental (KS and FL) populations. Thus, we set the pulse-pair rate to 95 s^−1^ and the peak amplitude to 80 dB SPL (0 dB = 20 µPa) in the high PR signal, and to 70 s^−1^ and 81 dB, respectively, in the high PA signal. For both signals, peak amplitude was measured in the center of the arena, the location where the test female was released. We adjusted the stimulus SPL to the predetermined values noted above with the aid of a sound pressure level meter (model CEL-430/2; Casella, Kempston, UK; flat frequency response from 30–20,000 Hz), confirmed with a calibrator (model CEL-110/2; Casella). We relied on the method of ‘peak equivalents’ to effect this adjustment by relating the millivolt output of a continuous 20 kHz broadcast, as measured by the condenser ultrasound microphone, to the SPL of this broadcast, as registered by the SPL meter. We then noted the millivolt output of the synthetic song stimulus broadcast as measured by the microphone, and we adjusted the gain on the loudspeaker amplifier until this millivolt output was equivalent to either 80 or 81 dB peSPL [27]. The lower PA value, 80 dB peSPL, was roughly equivalent to the song of a male *A. grisella* 10 cm distant, and it was 6–10 dB higher than average behavioral thresholds observed for female orientation toward male song. The positions of the loudspeakers broadcasting the two stimuli were switched on successive tests to preclude a side bias.

### Karyotyping

Because establishment of an accurate genetic map relied on a precise estimate of the number of expected linkage groups in *A. grisella*, we undertook a karyotypic analysis of this species. Here, we assumed that the haploid number of chromosomes observed would equal the number of linkage groups. We made our karyotypic analysis with fresh eggs taken from *A. grisella* females (Kansas population). Eggs were placed in a 1.5-ml tube and washed with a physiological solution (NaCl 0.9%). After centrifugation (400 g for 5 min) and elimination of the supernatant, eggs were placed in RPMI 1640 culture medium with colchicine (0.04 µg/ml final concentration), crushed with a piston pellet and incubated for 3 h at room temperature. The supernatant was then eliminated, and the pellet was resuspended in hypotonic solution (KCl 0.075 M) and incubated for 10 min at room temperature. Mitotic chromosomes were then fixed two times with a methanol: acetic acid solution (1∶1), spread on slides and, following DAPI staining, counted under a fluorescent microscope.

### Genotype analysis

#### AFLP markers

DNA extraction was performed with the DNeasy Tissue Kit (QIAGEN) according to the manufacturer's protocol, using bodies of adults stored at −80°C as starting material. DNA concentration and quality were assessed using a NanoDrop 1000 spectro-photometer (Thermo Scientific, Waltham, MA). AFLP markers were found in these DNA samples according to the method of Midamegbe et al. [42] but with some slight modifications: Following a double digestion of ∼100 ng of genomic DNA with EcoRI and MseI restriction enzymes, two successive selective PCRs were performed with the EcoRI+A primer

(5′-GACTGCGTACCAATTCA-3′) and either the Mse+C primer

(5′-GATGAGTCCTGAGTAAC-3′) or the Mse+G primer (5′-GATGAGTCCTGAGTAAG-3′) for the first PCR and the EcoRI+3 and Mse+3 primers for the second PCR (**Table 2**). Digestion/ligation mixes and PCR conditions were as in the method of Midamegbe et al. [42]. In total, 64 pairs of EcoRI+3 and Mse+3 primers were used. AFLP products were electrophoresed on an ABI 3130XL (Applied Biosystems, Foster City, CA) sequencer. Raw data were analyzed with GENEMAPPER© software (version 4.0), and individuals were scored for the presence or absence of any given AFLP marker. All genotypes were carefully confirmed by visual inspection, and we replicated some samples to avoid errors in AFLP scoring.

**Table 2:** List of primers used for the AFLP genotyping protocol, with the number of informative markers per brood. e = EcoRI (5′-GACTGCGTACCAATTC-3′) and m = Mse (5′-GATGAGTCCTGAGTAA-3′).

Primers	Broods		Primers	Broods	
	Xt7	Xt19		Xt7	Xt19
eAGA mCAC	19	8	eAGT mCAT	8	5
eAGT mCAA	13	9	eACA mCTC	8	3
eAGA mCAT	16	8	eACT mCGT	5	3
eAGA mCTA	15	9	eACC mGCC	14	8
eAGT mCTC	9	8	eACT mGCT	15	10
eATC mCAT	24	18	eACTmCGC	10	5
eATG mCAG	20	14	eATC mCGC	16	9
eATC mCAC	17	13	eATC mGAT	12	9
eACA mCAC	19	12	eACAmGCG	5	2
eATC mCAG	14	5	eATC mCTA	16	15
eACT mCAG	18	8	eATC mCGG	16	8
eACC mCTG	16	9	eATG mCTT	9	9
eACC mCAG	21	10	eACC mGTC	11	5
eACA mCAG	16	8	eATG mCAC	16	10
eACT mCTA	29	9	eACT mCTT	19	12
eACT mCTG	21	12	eACA mGAT	12	10
eATG mCGT	8	5	eACT mGCA	23	12
eATC mCGT	16	6	eACA mGCA	14	5
eACT mCGG	12	7	eATC mGCG	8	7
eATC mGCC	13	8	eACG mGTC	13	7
eACG mCAG	14	10	eACCmCAC	-	13
eACA mCTG	11	9	eACAmGAA	-	7
eACT mCAC	12	8	eACAmCAT	-	7
eACA mCGC	14	7	eACAmGTA	-	8
eACT mGAA	11	11	eAGAmCAA	-	9
eACG mCTG	12	9	eACGmCTC	-	3
eACC mCTA	17	10	eACTmCAT	-	5
eACA mCTA	11	11	eACTmCGC	-	7
eATC mGCA	12	6	eACTmCAA	-	7
eACA mGTC	14	5	eATGmCAT	-	6
eACT mGCC	13	5	eAGAmCAG	-	7
eACG mCGG	6	6	eAGAmCGT	-	2

#### Construction of genetic maps

Based on the nature of AFLP polymorphism and the crossing design, we constructed one map for each cross using the 1∶1 segregating markers present in the F1 male parent (+/−), resulting from a cross between a Kansas female and a Florida male, and absent in the pure Kansas female parent (−/−). These maps will be referred as Xt7 and Xt19 maps, following the identification code of their respective broods. Linkage analysis was performed with the Joinmap®4 software [43]. First, chi-square tests (χ^2^) were performed on each AFLP marker for goodness of fit to the expected Mendelian 1∶1 segregation ratio. Loci that deviated significantly (*P*<0.001) from this ratio were judged as ‘distorted’ and excluded from the map construction. Linkage groups were identified based on a LOD score (logarithm_10_ of odds) of 6. The ordering of the markers within linkage groups was determined by a modified least squares procedure using the default parameter values of JoinMap®4.0 except for the goodness-of-fit ‘jump threshold’ for removal of loci. This latter parameter was set to 3.0, which is a more stringent value than the default one. Recombination values were converted into map distances (in cM) by applying the Kosambi mapping function [44].

#### QTL analysis

QTL analysis was carried out using numerical values for all parameters. The analyzed traits are listed in **Table 1**. Computational analysis was carried out using MapQTL®5 software [45]. A first preliminary analysis was performed using a nonparametric mapping method (Kruskal-Wallis rank sum test) to detect simple marker/trait association. We then applied the interval mapping algorithm [46] and selected the QTL position with the highest LOD score for each trait. The LOD score threshold of significance was estimated by the resampling techniques outlined in Churchill & Doerge [47] and was based on 1000 permutations. We ran a final analysis using composite interval mapping (CIM) with a maximum of 5 cofactors to account for the possibility that several QTLs might be segregating in the populations. These cofactors represented the nearest markers, detected with the nonparametric mapping method, to each QTL. The confidence interval for each QTL was calculated by finding the locations on either side of its peak that corresponded to a decrease in the LOD score by 1 unit.

## Results

### Karyotyping

We observed a haploid number of 30 chromosomes in 6 of the 10 microscope slides prepared, and slightly smaller haploid numbers in the remaining 4 slides. Because of the difficulty in spreading the small chromosomes in *A. grisella*, we considered that the actual number was likely 30, while the observations of lower counts possibly reflected incomplete spreading.

### Construction of the two linkage maps

733 and 518 AFLP markers were analyzed for the Xt7 and Xt19 broods, respectively. Because of the segregation distortion of some of these markers and the occurrence of unmapped markers, a final count of 442 and 241 AFLP markers were positioned on the Xt7 and Xt19 maps, respectively. Thirty-three linkage groups were obtained for Xt7 and 32 for Xt19 at a LOD score of 6. These numbers of linkage groups determined in the two broods are largely consistent with our cytological observations. We note that numbers of linkage groups slightly exceed the haploid number of chromosomes observed (n = 30), a discrepancy that may be due to the conservative (high) LOD we used and to the incomplete saturation of the linkage maps.

The total lengths of the linkage maps were 1390 cM for Xt7 and 599 cM for Xt19, with linkage group length ranging from 3.0 cM to 86.5 cM for Xt7 and from 1.0 cM to 57.0 cM for Xt19. The average interval distance between two consecutive markers was 3.4 cM for Xt7 and 2.9 cM for Xt19. The markers were not evenly distributed among the linkage groups. Some regions exhibit clusters of tightly linked markers, while others have gaps greater than 10 cM between consecutive markers.

Most markers were not informative in one of the two maps, and only 75 markers among the 608 markers observed in total were common to both Xt7 and Xt19. Based on these common markers, 23 pairs of linkage groups were associated between Xt7 and Xt19. At least two common markers are needed to determine the relative orientation of two linkage groups. This criterion was satisfied in 14 of the pairs of associated linkage groups, thus revealing their relative orientation. When two or more markers were present in a linkage group in a cross, the marker order was conserved for 12 of these 14 pairs of linkage groups, except for several differences that involved tightly linked markers (distances below 4 cM). Because of the small sample sizes of the populations, minor differences in recombination fraction could translate to slightly different orders between the two populations. The marker order differed more strongly for the 2 remaining pairs of associated linkage groups that had 2 or more common markers. Here, either one marker did not follow the real order, or inversion of chromosome segments occurred. These discrepancies occurred in the association of LG 3 (linkage group 3) from Xt7 and LG2 from Xt19 and for LG 16 from Xt7 and LG 10 from Xt19 (**Figure S1, sections 3 and 13**).

### Trait distribution in the broods

**Figure 1:**
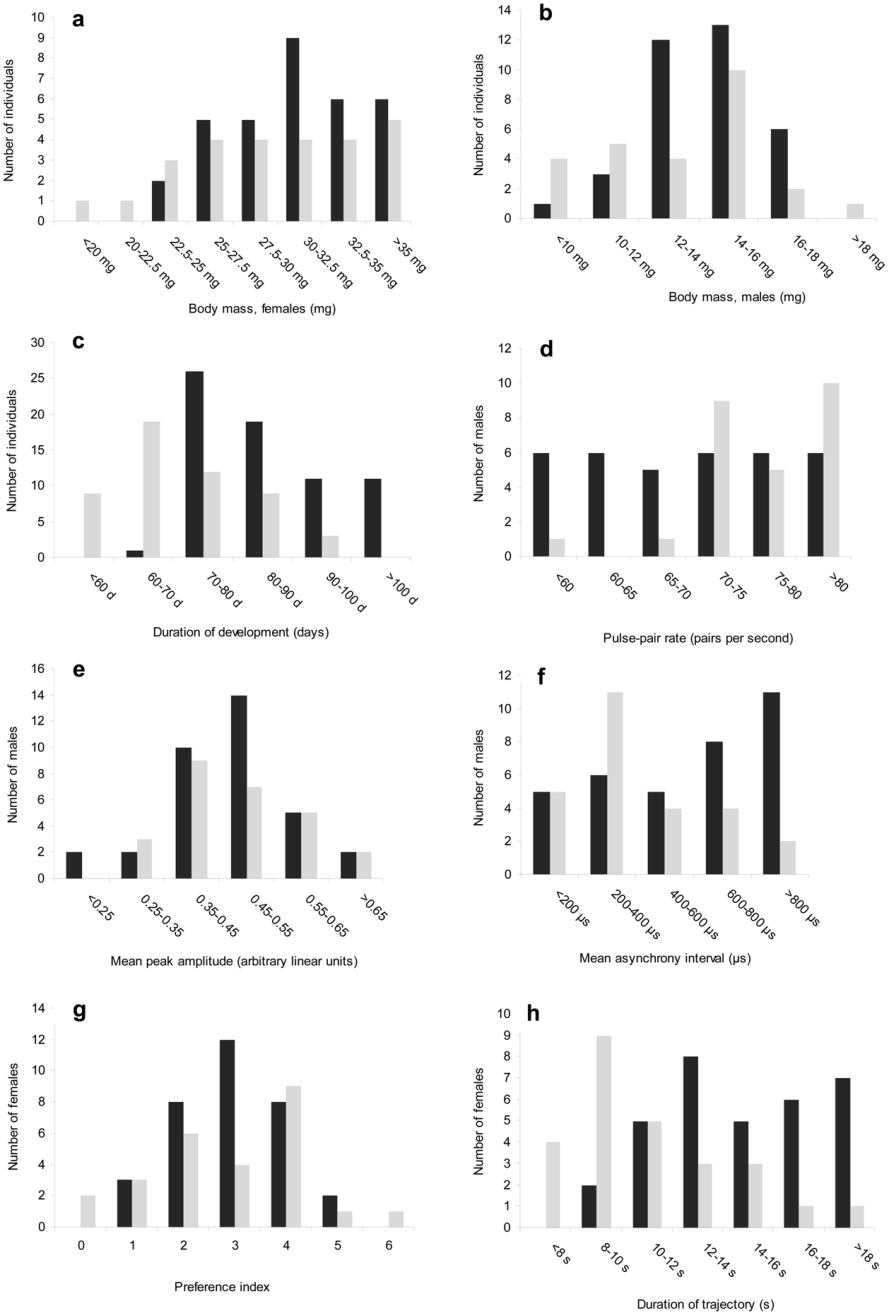
Phenotypic variation of developmental and sexual traits observed in both broods sampled for genotype analysis. In each graph, the dark and light vertical bars represent brood Xt7 and brood Xt19, respectively. CV_7_ and CV_19_ indicate the coefficients of variation for brood Xt7 and brood Xt19, respectively. t-test (2-tailed) as applied to within-brood comparisons where data satisfied the requirements of normality and equality of variance; H_0_: average_Xt7_ = average_Xt19_. Mann-Whitney test (2-tailed) as applied to within-brood comparisons where data did not satisfy the requirements of normality and equality of variance; H_0_: average_Xt7_ = average_Xt19_. (A) Body mass at adult eclosion, females. (B) Body mass at adult eclosion, males. (C) Duration of development of tested individuals. (D) Pulse-pair rate of male song. (E) Mean peak amplitude of male song. (F) Mean asynchrony interval duration of male song. (G) Female preference index. (H) Duration of female trajectory from release point to arrival at one of 2 stimuli (broadcasting loudspeakers).

Phenotypic variation, necessary for QTL analyses, was observed for all measured developmental and sexual traits in both broods (**Figure 1**). We found that female mass was approximately the same in the two broods, with average values of 31.03 mg (range from 23.81 to 36.65 mg) in Xt7 and 30.32 mg (range from 18.48 to 43.74 mg) in Xt19 (**Figure 1a**). For male mass, we also observed similar values in the two broods, with a mean of 14.16 mg (range from 9.72 to 17.49 mg) in Xt7 and a mean of 13.19 mg (range from 7.89 to 18.12 mg) in Xt19 (**Figure 1b**). However, average developmental time was longer in Xt7 (mean of 86 d; range from 69 to 119 d) than in Xt19 (mean of 71 d; range from 52 to 93 d), (Mann-Whitney test, *P*<0.001) (**Figure 1c**). The mean pulse-pair rate of male song was significantly lower in Xt7 (range from 54 to 83 s^−1^) than in Xt19 (range from 59 to 89 s^−1^), (t-test, *P*<0.001) (**Figure 1d**). Mean peak amplitude ranged from 0.234 to 0.704 (arbitrary linear units) and from 0.255 to 0.687 in Xt7 and Xt19, respectively (**Figure 1e**). Mean asynchrony interval varied from 0 to 2904 µs in Xt7 and from 0 to 1135 µs in Xt19, and was significantly higher in Xt7, (M-W test, *P* = 0.018) (**Figure 1f**). For female preference, we observed that some individuals oriented preferentially toward the signal with a low PR and a high amplitude, whereas others preferred the signal with a high PR and a low amplitude. Thus, the preference index ranged from 0 (the female chose the high PR signal in all 6 tests) to 6 (the female chose the high PA signal in all 6 tests), with a mean of 2.85 in Xt19 and from 1 to 5, with a mean of 2.94 in Xt7 (**Figure 1g**). In arriving at one of the two stimuli, females spent on average more time in Xt7 (mean of 15.6 s; range from 9 to 36.8 s) than in Xt19 (mean of 11.3 s; range from 6.5 to 31.8 s), (M-W test, *P*<0.001), (**Figure 1h**). For all characters, we observed a unimodal distribution for the measured trait.

### QTL detection

**Figure 2:**
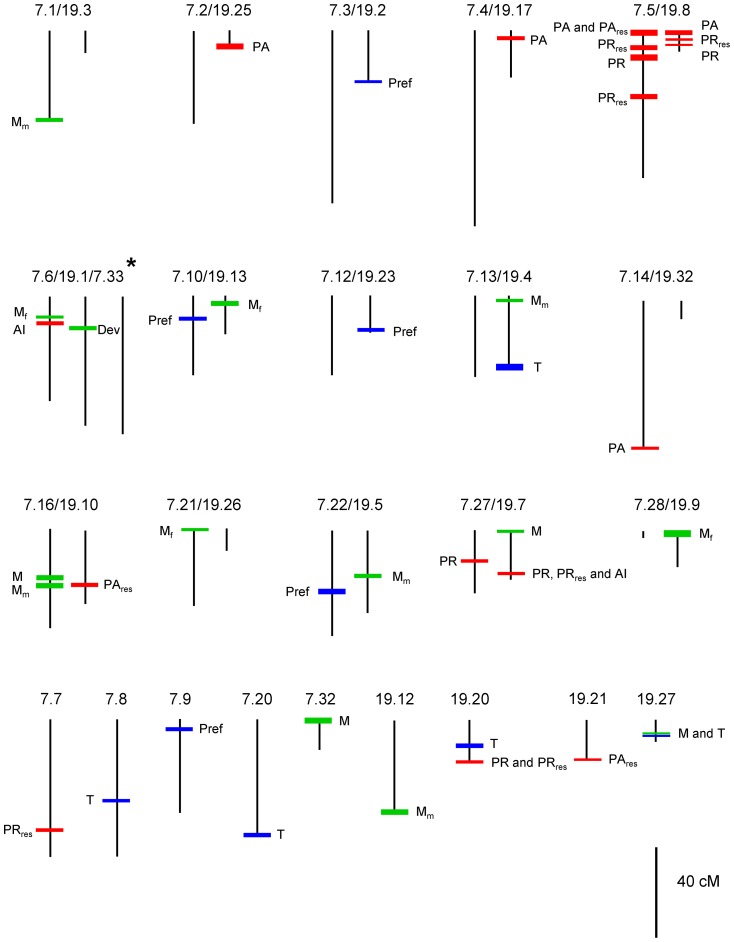
Distribution map for QTLs detected for developmental, male signal, and female receiver traits. Based on common markers in broods Xt7 and Xt19, homologous linkage groups were determined for the two broods, and the pairing of these homologous groups is represented by parallel vertical lines. Unpaired lines represent cases where a lack of common markers prevented determination of homologous linkage groups. Because common markers indicated a link between linkage group 1 in Xt19 (19.1) and two linkage groups, 6 and 33, in Xt7 (7.6 and 7.33), three parallel lines are shown in this particular case.* The colored, horizontal line indicates the position of the QTL, and its thickness is proportional to the LOD score. Green, red and blue horizontal lines represent developmental, male signal, and female receiver traits, respectively. Linkage group identities (brood . linkage group number) are shown above each graph, and trait names are listed next to the horizontal line representing each QTL. Map distances are in cM, estimated by the Kosambi mapping function. Paired and unpaired linkage groups in which we did not detect any QTLs are not represented in this figure.

To detect QTLs, we successively used single marker analysis, interval mapping, and composite interval mapping (CIM), which were performed separately for the two broods. Because the results of the three methods were similar, we only present CIM results here. Significant QTLs (LOD score>1.8, except for four QTLs with scores of 1.3 and 1.6) were detected in both broods Xt7 and Xt19 for each developmental and sexual trait that we measured. In total, we found 20 and 25 QTLs in Xt7 and Xt19, respectively (**Table 3** **,** **Table 4**). Owing to the relatively large number of linkage groups, only linkage groups on which QTLs were detected are illustrated on **Figure 2**. Maps for the other linkage groups are given in detail in **Figure S1**.

**Table 3:** QTLs detected for developmental, male signal, and female receiver traits measured in brood Xt7.

Trait Code	LG	QTL Name	Marker Interval	D	LOD	Additive Effect	R^2^ (%)
M	16	M7.1	eACGmCAG_369.38–eACAmCTA_164.32	24	3.2	−4.13	22
M	32	M7.2	eAGAmCAC_263.29–eAGAmCAC_400.16	0	3.7	−4.62	23.4
M_m_	1	M_m_7.1	eAGAmCAC_420.65–PeAGAmCTA_200.16	39.4	2.5	0.87	20.2
M_m_	16	M_m_7.2	eACAmCGC_176.51–eACAmCTA_164.32	29.9	3.4	−1.19	32
M_f_	6	M_f_7.1	PeAGAmCTA_267.11–PeACTmCTT_130.66	9.8	1.9	1.76	23.3
M_f_	21	M_f_7.2	eAGTmCAA_128.47–PeACTmGAA_135.45	0	2.0	−1.80	24.4
PR	5	**PR7.1**	eATCmCGT_268.73–PeACTmCAC_166.77	16	4.0	−6.42	**51.7**
PR	27	PR7.2	eACAmGCA_468.45–eATCmCAG_173.37	13.7	2.2	3.85	18.9
PR_res_	5	PR_res_7.1	eATCmCGT_268.73–PeATGmCAC_148.04	11	3.1	−6.40	46.5
PR_res_	5	PR_res_7.2	eACCmCAG_116.37–PeATCmCGG_307.86	30.6	3.3	−5.60	40.7
PR_res_	7	PR_res_7.3	eACAmGCA_139.58–eAGAmCTA_118.89	51.8	2.3	−4.81	29
PA	5	PA7.1	eATCmCGT_268.73–PeATGmCAC_148.04	0	3.8	−0.085287	35.1
PA	14	PA7.2	eATCmCGC_210.37–PeACAmGAT_397.05	65.6	1.9	−0.042400	15.2
PA_res_	5	PA_res_7.1	eATCmCGT_268.73–PeATGmCAC_148.04	0	2.1	−0.065765	23.9
AI	6	AI7.1	PeACCmCTG_350.45–PeACTmCTT_130.66	11.8	2.6	936.77	26.5
Pref	9	Pref7.1	eATCmGCC_110.75–eATCmGCA_351.33	6.1	2.5	0.48	18.4
Pref	10	Pref7.2	PeACTmCTA_306.69–eACTmCGT_87.09	12.1	2.5	0.45	18.4
T	22	Pref7.3	eACTmCAC_182.31–eAGTmCAA_111.34	27.3	3.5	−0.57	27.4
T	8	T7.1	eACCmGCC_243.35–eATCmGAT_103.35	36.8	2.1	−2.13	26.3
T	20	T7.2	eACGmCTG_317.64–eACCmCAG_313.36	51.6	2.7	−2.42	32.3

See Table 1 for trait descriptions.

Main effect QTLs determined via CIM mapping in Xt7, with additive effect (the estimated additive effect of the QTL is an estimate of the change in the average phenotype that would be produced by substituting a single allele of one type with that of another type) and R^2^ ( = proportion of phenotypic variance explained by the QTL after accounting for co-factors) shown in the two columns at the right. LG indicates the linkage group where the QTL is situated, marker interval is delimited by the two AFLP markers enclosing the QTL, and D is the distance, measured in cM, from the telomere to the QTL. PA_res_ and PR_res_ are the residual values.

**Table 4:** QTLs detected for developmental, male signal, and female receiver traits measured in brood Xt19.

Trait Code	LG	QTL Name	Marker Interval	D	LOD	Additive Effect	R^2^ (%)
Dev	1	Dev19.1	PeACCmGCC_152.01–PeATCmCAT_222.42	12.3	2.6	−4.81	20.7
M	7	M19.1	eACAmCAC_179.37 – eACTmGCT_296.75	0	1.9	3.94	15.8
M	27	M19.2	eACAmGAA_104.2–eACTmGCA_180.74	5.9	1.9	3.94	15.8
M_m_	4	M_m_19.1	PeATGmCAC_136.75–PeACAmGTC_430.99	3.9	2.1	1.21	17.6
M_m_	5	M_m_19.2	eACTmCGC_214.53–eAGAmCAT_231.11	19.3	2.6	−1.37	24.7
M_m_	12	M_m_19.3	eATCmGCA_329.7–eACTmCTT_113.92	40.3	3.5	−1.70	34.8
M_f_	9	M_f_19.1	PeACCmCTG_132.77–PeATCmCGC_132.74	0	4.4	4.72	46.7
M_f_	13	M_f_19.2	eACCmCAC_238.25–PeACTmCTA_306.69	3.1	3.1	3.50	29.5
PR	7	PR19.1	eACAmCAC_179.37–eATCmGAT_310.62	18.5	1.3	−2.97	20.9
PR	8	PR19.2	PeATGmCAC_148.04–PeATGmCTT_205.56	5.8	1.3	2.88	19.9
PR	20	PR19.3	eeACAmCAC_430.21–eACAmGTA_120.0	18.6	1.9	3.66	28.9
PR_res_	7	PR_res_19.1	eACGmCTG_116.14–eATCmGAT_310.62	18.5	1.9	−3.30	28.1
PR_res_	8	PR_res_19.2	PeATGmCAC_148.04–PeATGmCTT_205.56	3.5	1.6	3.11	25.2
PR_res_	20	PR_res_19.3	eeACAmCAC_430.21–eACAmGTA_120.0	18.6	2.2	3.67	31.7
PA	8	PA19.1	PeATGmCAC_148.04–PeACTmCAC_166.77	0	3.0	0.053899	21.7
PA	17	PA19.2	PeACTmCTG_170.82–PeATCmGCC_93.41	3,1	2.5	−0.046967	16.9
PA	25	PA19.3	eATCmGAT_127.65–eAGTmCAA_116.97	7.3	3.8	0.058194	28.8
PA_res_	10	PA_res_19.1	PeACTmGCT_163.05–eATGmCAT_129.31	24.3	2.6	0.054276	33.1
PA_res_	21	PA_res_19.2	eACCmGCC_104.7–eAGAmCAT_95.9	17.9	1.6	0.040841	18.7
AI	7	AI19.1	eAGTmCAA_318.11–eATCmGAT_310.62	18.5	2.7	179.75	37.5
Pref	2	Pref19.1	PeACAmCAC_133.45–PeACTmGCA_241.6	22.6	1.8	0.63	17.8
Pref	23	Pref19.2	eACTmCAT_327.57–eACGmCTC_439.07	16.3	2.5	−0.82	26.1
T	4	T19.1	eACCmCTA_369.26–PeACCmGCC_123.82	30.9	4.2	−2.22	43.4
T	20	T19.2	eeACAmCAC_430.21–PeACTmCAG_257.47	11	2.8	−1.63	26.6
T	27	T19.3	eACAmGAA_104.2–eACTmGCA_180.74	5.9	3.3	−1.87	31.2

See Table 1 for trait descriptions.

Main effect QTLs determined via CIM mapping in Xt19, with additive effect (the estimated additive effect of the QTL is an estimate of the change in the average phenotype that would be produced by substituting a single allele of one type with that of another type) and R^2^ ( = proportion of phenotypic variance explained by the QTL after accounting for co-factors) shown in the two columns at the right. LG indicates the linkage group where the QTL is situated, marker interval is delimited by the two AFLP markers enclosing the QTL, and D is the distance, measured in cM, from the telomere to the QTL. PA_res_ and PR_res_ are the residual values.

We detected QTLs for the several developmental traits measured, notably for body mass. For development time, we found only one QTL located on LG1 and explaining 20.7% of the phenotypic variation in brood Xt19. For male body mass we found two QTLs in Xt7 and three in Xt19. Similarly, for female body mass we found two QTLs in both broods. All of the QTLs for body mass were localized on independent linkage groups, and they explained from 20.2% to 32% of the phenotypic variation in Xt7 and from 15.8% to 46.7% in Xt19.

For the male signal traits, we found independent QTLs positioned on different linkage groups for all three signal characters measured. We found only one QTL for asynchrony interval, which was detected in each brood and explained 26.5% and 37.5% of the variation in Xt7 and Xt19, respectively. We identified two and three QTLs for peak amplitude in broods Xt7 and Xt19, respectively. These QTLs explained between 15.2 and 35.1% of the variation. Finally, we identified two and three QTLs for pulse-pair rate in broods Xt7 and Xt19, respectively. These QTLs explained 18.9 and 51.7% of the variation in Xt7 and from 19.9% to 28.9% of the variation in Xt19. In analyzing the residual values of peak amplitude or pulse-pair rate (PA_res_, PR_res_) in the linear regressions of these parameters on M_m_, we found some QTLs (PR_res_7.1, PR_res_7.2, PA_res_7.1) that may be identical to those found for these two song characters (PA, PR) as well as one new QTL (PR_res_7.3) (see **Table 3**). Importantly, the QTLs identified for peak amplitude (PA) and pulse-pair rate (PR) were not found on the same linkage groups as the QTLs for M_m_ in either brood (**Table 3** **,** **Table 4** **,** **Figure 2**). Thus, to the extent that the QTLs for peak amplitude and pulse-pair rate are valid, they reflect male song rather than artifacts of body mass. Details on all identified QTLs are given in **Table 3** **,** **Table 4** and **Figure S1**.

We also found QTLs for the female preference index on different linkage groups. In brood Xt7, three QTLs were observed on LG 9, LG 10 and LG 22, respectively, and these explained between 18.4 and 27.4% of the variation in the preference index. In brood Xt19, two QTLs were found on LG 2 and LG 23, explaining 17.8 and 26.1% of the variation, respectively. We detected two and three QTLs for the time spent reaching a chosen song stimulus in broods Xt7 and Xt19, respectively. These different QTLs explained from 26.3 to 43.4% of the phenotypic variation for this female response trait.

The bridges established between 23 of the 33 linkage groups of Xt7 and Xt19 based on markers common to both linkage maps allowed us to examine the relative locations of QTLs in the two broods. For a given trait, most of the QTLs were specific to one brood, but a few were found at homologous positions in both broods. We observed three QTLs for male song traits (two QTLs for PR and one QTL for PA) that were located on homologous linkage groups in both Xt7 and Xt19. Thus, QTLs for pulse-pair rate (PR) were found on syntenic positions on LG 5 (PR 7.1) and LG 8 (PR 19.2) in Xt7 and Xt19, respectively, and another pair of QTLs for PR was found on syntenic positions on LG 27 (PR7.2) and on LG 7 (PR 19.1) in Xt7 and Xt19, respectively. For peak amplitude (PA), we found QTLs on syntenic positions on LG 5 (PA 7.1) and LG 8 (PA 19.1) in Xt7 and Xt19, respectively (**Figure 2**).

Co-localization on the same linkage group was also observed for different male song traits. Two QTLs for PR and PA were situated in similar positions on homologous LGs in Xt7 and Xt19, and consequently these two QTL may be linked to a male signal parameter that could be characterized as the acoustic power of the signal (**Figure 2**). In contrast, we observed no co-localization between male signal traits and female preference traits. We also observed no co-localization between male song traits and male body mass, as well as between female preference traits and female body mass.

## Discussion

### QTLs for signal, receiver, and developmental traits

Our analyses indicated QTLs of at least moderate influence (LOD score≥1.8) for all of the male song, female response and preference (receiver traits), and developmental traits that we measured, and three QTLs that exerted a major influence (LOD score≥4.0) on the pulse-pair rate in male song (PR 7.1), the speed of female response (T 19.1), and female body mass (M_f_ 19.1), respectively (**Table 3** **,** **Table 4**). Several QTLs were detected for most traits. Comparable numbers of QTLs were found in the two broods, designated Xt7 and brood Xt19. The QTLs identified in this study were distributed among more than 20 of the 30 linkage groups in the *A. grisella* genome, and we did not find any obvious clustering of QTLs in certain groups (chromosomes), either for all traits or for any of the three trait categories. Eight of the QTLs identified in Xt7 and four of the QTLs identified in Xt19 had LOD scores>3.0. In Xt7 these included QTLs influencing two male song traits (pulse-pair rate and peak amplitude), one female receiver trait (preference index), and two developmental traits (body mass of all individuals, male body mass). These values are comparable to those found in other QTL studies similarly constrained by low sample sizes owing to difficult phenotyping performed on complex behavioral traits performed on non-model organisms [48]–[50].

Several QTLs were found in syntenic positions in the two broods. Two of the male song traits, pulse-pair rate and peak amplitude, were each associated with QTLs located on syntenic positions in Xt7 and Xt19 (PR 7.1, PR 19.2; PA 7.1, PA 19.1; **Figure 2**), a situation rendering our inferences about these specific QTLs particularly reliable. Notably, one of these QTLs (PR 7.1) found in syntenic positions in the two broods is the one that had a major influence on pulse-pair rate in Xt7, having a LOD score of 4.0 and explaining 51.7% of the phenotypic variance (**Table 3** **,** **Figure 2**). This finding is of particular importance because pulse-pair rate, being subject to directional female preference [27] and uncorrelated (in the populations we studied) with body size, is unambiguously a sexually-selected trait. Moreover, the high influence of this QTL is corroborated by an earlier artificial selection study in which lines for fast and slow pulse-pair rates were developed after only 3–5 generations of selection [51]. Separation of lines in so few generations would not be expected for a trait whose expression is largely under polygenic influence. Possibly, the pulse-pair rate QTL owes its strength to two parallel influences, female preference and predation by insectivorous bats – which has also selected for faster pulse rates that the moths would not confuse with echolocation signals of their predators [41].

The number and effects of QTLs observed in our study have to be interpreted within the context of the relatively small populations that we sampled. Small sample sizes such as ours are regularly encountered in studies of non-model organisms subject to strong experimental constraints, and they may reduce an ability to detect QTLs and artificially inflate estimates of the effects of the individual QTLs that we did detect, the so-called ‘Beavis effect’ [52], [53]. Nevertheless, our data on the overall percentage of phenotypic variation explained by the various QTLs (**Table 3** **,** **Table 4**) suggest that most traits are influenced polygenically. The remaining variation, unaccounted for by the identified QTLs, may be explained by environmental influence (i.e. inevitable differences between different rearing containers), QTLs not detected in this study, and genetic factors having undetectable effects in our specific experimental design (genetic background of tested individuals, sample sizes). Similar levels of polygenic influence have been observed in other QTL studies on song traits in acoustic species [15], [54] and may be a general feature of acoustic communication [55].

### On the absence of co-localization

Despite finding QTLs for the several male song and female response traits tested, we did not observe any co-localization of song and response QTLs on the same linkage groups in either brood. While male song characters were analyzed rather thoroughly, we scored only two female receiver traits, the latency or duration of a female's trajectory toward a male song stimulus and her relative weighting of pulse-pair rate vs. peak amplitude in evaluation of male song. Although we designed the test for the second trait based on observed variation in male song and our previous selection gradient studies on female evaluation of song [28], other female receiver traits, e.g. the shape and steepness of a preference function for a single character such as pulse-pair rate, may also be critical. That is, absence of evidence does not automatically imply that co-localization does not exist.

Second, it is possible that a larger sample of backcross generation females would have revealed other QTLs for the preference trait (Pref), some being localized on the same linkage groups as QTLs for corresponding male song traits (PR, PA). While we recognize this potential problem – and possibility – we also note the large number of informative markers used to develop our linkage map and the robustness of many of the identified QTLs, including one for female preference (Pref 7.3, which accounts for 27.4% of the phenotypic variance and has a LOD score of 3.5). A larger sample, not suffering from the Beavis effect, might also indicate weaker influences of identified QTLs than the values listed in **Table 3** and **Table 4**. But this reduction in influence would be expected to affect all values such that the QTLs listed with the highest influences might still have actual values in the moderate range. Because we observed no tendency for QTLs currently listed with major influences to be co-localized, we suggest that an increased sample size might not inevitably yield fundamentally different results pertaining to the issue of co-localization of male song and female receiver QTLs.

Third, as may always occur in QTL mapping studies, the observed number, positions and effects of QTLs can be specific for the parental lines analyzed, and other results might be forthcoming in a different genetic background [56], [57]. This possibility does not necessarily negate our findings discussed above but rather suggests that more studies would be needed before arriving at a general and definitive conclusion for *A. grisella*.

Finally, assuming that the results of our QTL analysis were not strongly biased by the samples and methods we employed, the observed absence of co-localization may actually be representative of sexually selected traits in *A. grisella* as well as among animal species in general. This possibility demands a review of other studies and a different view of the sexual selection process as it occurs in natural populations, both of which we provide in the following section.

### On the tempo of sexual selection in natural populations

Over the past 20 years biologists have tested the expectation that indirect benefits mechanisms of sexual selection generate or are associated with a genetic covariance [58] between male signal and female receiver traits. These studies have relied on the methods of quantitative genetics to assess whether signal and receiver traits covary, and some have employed specific breeding designs (e.g. full sib/half sib) to yield accurate measures of covariance [59]. While several of the earlier studies reported evidence for genetic covariance [60]–[62], the vast majority of the studies, conducted on various invertebrate [63], [64] and vertebrate species [65], have not (see [66] for review). Some of these studies may have failed to detect genetic covariance because a procedure of random pairing employed in the laboratory would have greatly reduced the linkage disequilibrium between male signal and female preference traits that had existed in the field owing to non-random mate choice [6]. But several of the studies that did not reveal genetic covariance did take appropriate steps to avoid this potential difficulty by estimating covariance in the field or immediately after the collection of a field population [67], [68]. In particular, one of these studies was on *A. grisella*, where a potential genetic covariance between the pulse-pair rate in male song and the threshold rate eliciting female orientation and phonotaxis was investigated [66]. Whereas the objective of these studies was a determination of total genetic covariance between male signal and female receiver traits without regard to its origin, this total value would have included the covariance originating from pleiotropy or tight physical linkage. Thus, the overall evidence does not support a genetic architecture in which factors influencing male signal and female receiver traits are co-localized.

The general expectation of genetic covariance, due to linkage disequilibrium from either non-random mating or physical linkage, between male signal and female receiver traits assumes a certain ‘strength of sexual selection’ [7], [8], a strength level that generates exaggerated traits and maintains this pressure more or less continuously. There currently exists some controversy on the appropriate method for measuring the strength of sexual selection in natural populations [69], and yet more controversy on whether sexual selection is actually a significant factor that shapes traits in males and females [70]. While a prevailing view is that sexual selection does represent a potent force [1], it is possible that this potency arises only occasionally during the course of evolution. If so, we may normally observe signal and receiver traits that are more or less in an equilibrium state during which their trajectory toward greater exaggeration is markedly tempered. Additionally, male signals and female preferences are generally complex traits that represent the composite of multiple characters, each subject to its own polygenic influence. Often, the several characters comprising a signal covary themselves, and a factor that selects for exaggeration of one character may inevitably select for diminution of another. Under the various conditions above we have no reason to expect a consistent amount of genetic covariance between male signal and female receiver traits, and an absence of covariance is what has generally been observed (see [71] for review). Thus, an accelerated tempo of sexual selection driven by signal/receiver genetic covariance may be more of an exception than the norm in natural populations. In *A. grisella* this prediction may imply that selection imposed by female choice on male signal traits is usually relatively weak except during certain episodes, as when genotype×environment interaction decreases and allows linkage disequilibrium due to mate choice to rise temporarily. Otherwise, the expression of male signal traits such as pulse-pair rate may remain stable and subject to balancing pressures of species recognition, natural selection, and mate choice.

## Supporting Information

Figure S1 **This file illustrates the mapping of QTLs for developmental, male signal, and female receiver traits among linkage groups in broods Xt7 and Xt19.** Each section of the file shows maps of a pair of associated linkage groups where association was possible due to common markers, or only one linkage group where common markers did not occur. Two sections (6 and 7) show three linkage groups because linkage group 1 in Xt19 was associated with two linkage groups, 6 and 33, in Xt7, and linkage group 6 in Xt19 was associated with two linkage groups, 7 and 17, in Xt7. For each linkage group map in every section, AFLP markers are listed on the right and their locations, measured in cM (estimated by the Kosambi mapping function) from the telomere, are shown on the left. Lines that connect the maps of associated linkage groups indicate the common markers. Solid triangles to the right of a linkage group map indicate the position of a detected QTL, with triangles pointing upward and downward representing QTLs that exert positive and negative effects, respectively, on the value of a given trait. Triangle size is proportional to the LOD score, the thick vertical line represents the confidence interval (locations on either side of the QTL at which the LOD score decreases by 1 unit relative to the peak) for location, and the QTL name is listed at the top of this line (see Tables 3 and 4 for corresponding information).
(PDF)
