# Supplementary material for: Genetic Architecture of Sexual Selection: QTL Mapping of Male Song and Female Receiver Traits in an Acoustic Moth
Source: PLoS One. 2012 Sep 5;7(9):e44554. doi: 10.1371/journal.pone.0044554 (PMC3434148; doi:10.1371/journal.pone.0044554)

Section 1. Linkage group 1 in brood Xt7 and linkage group 3 in brood Xt19; association indicated by common markers.

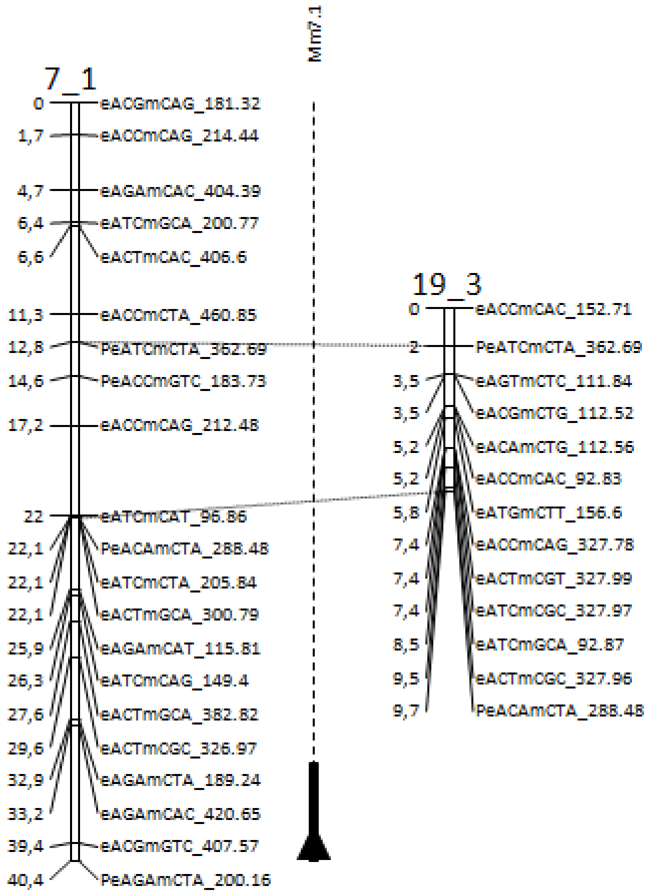

Section 2. Linkage group 2 in brood Xt7 and linkage group 25 in brood Xt19; association indicated by common markers.

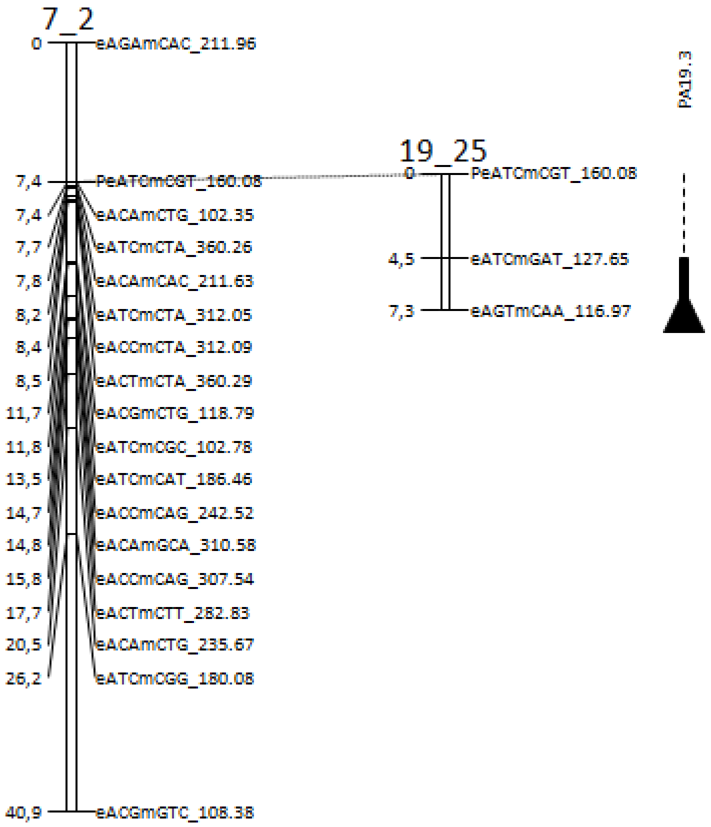

Section 3. Linkage group 3 in brood Xt7 and linkage group 2 in brood Xt19; association indicated by common markers.

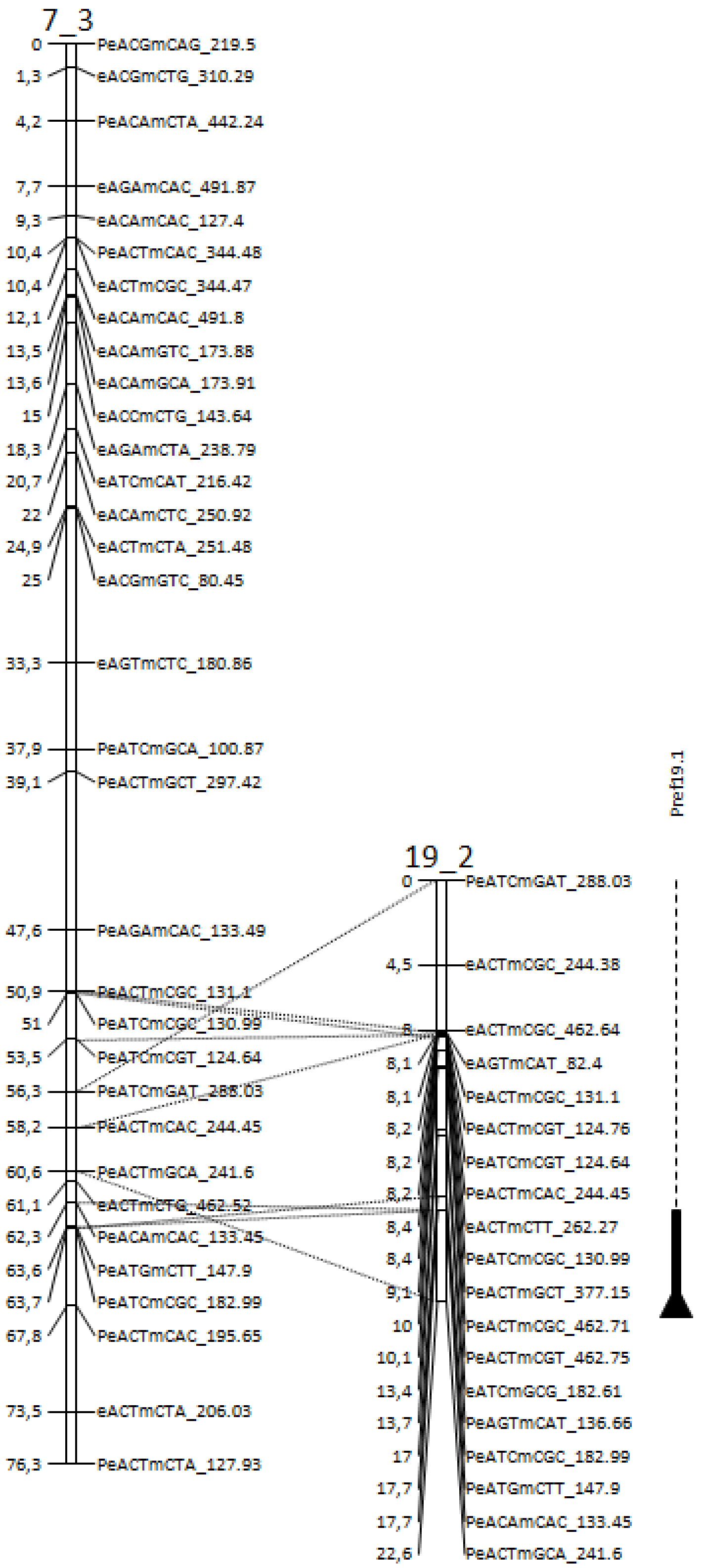

Section 4. Linkage group 4 in brood Xt7 and linkage group 17 in brood Xt19; association indicated by common markers.

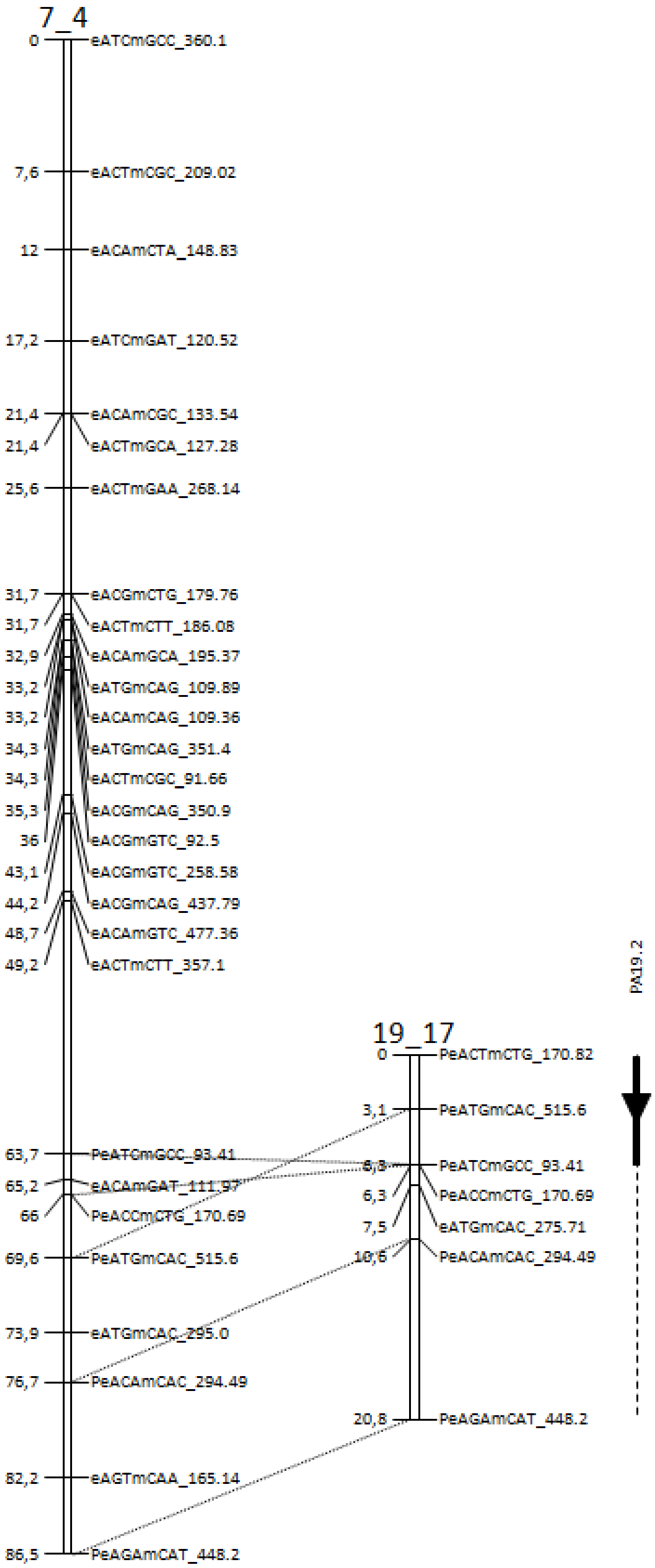

Section 5. Linkage group 5 in brood Xt7 and linkage group 8 in brood Xt19; association indicated by common markers.

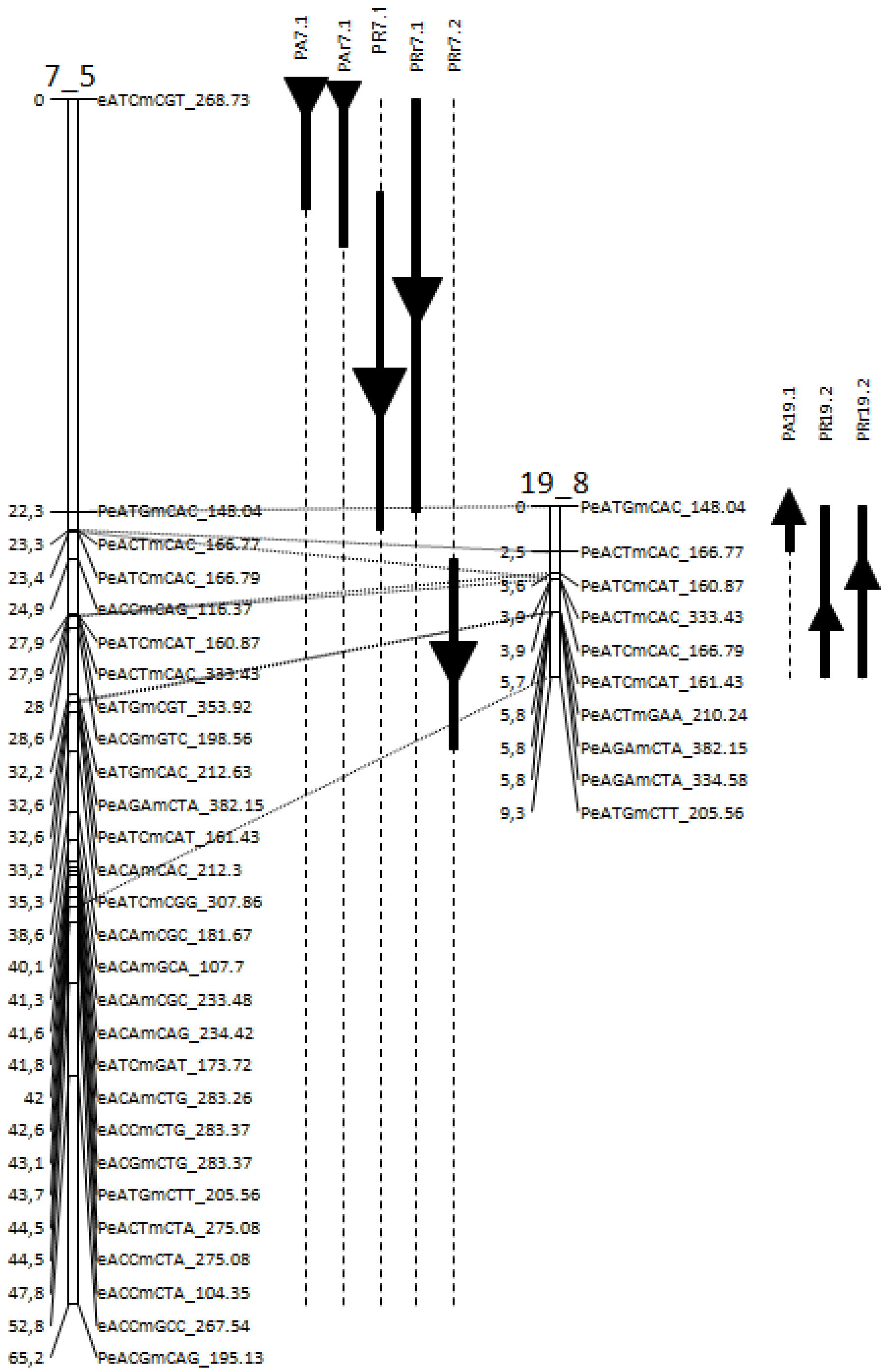

Section 6. Linkage groups 6 and 33 in brood Xt7 and linkage group 1 in brood Xt19; association indicated by common markers.

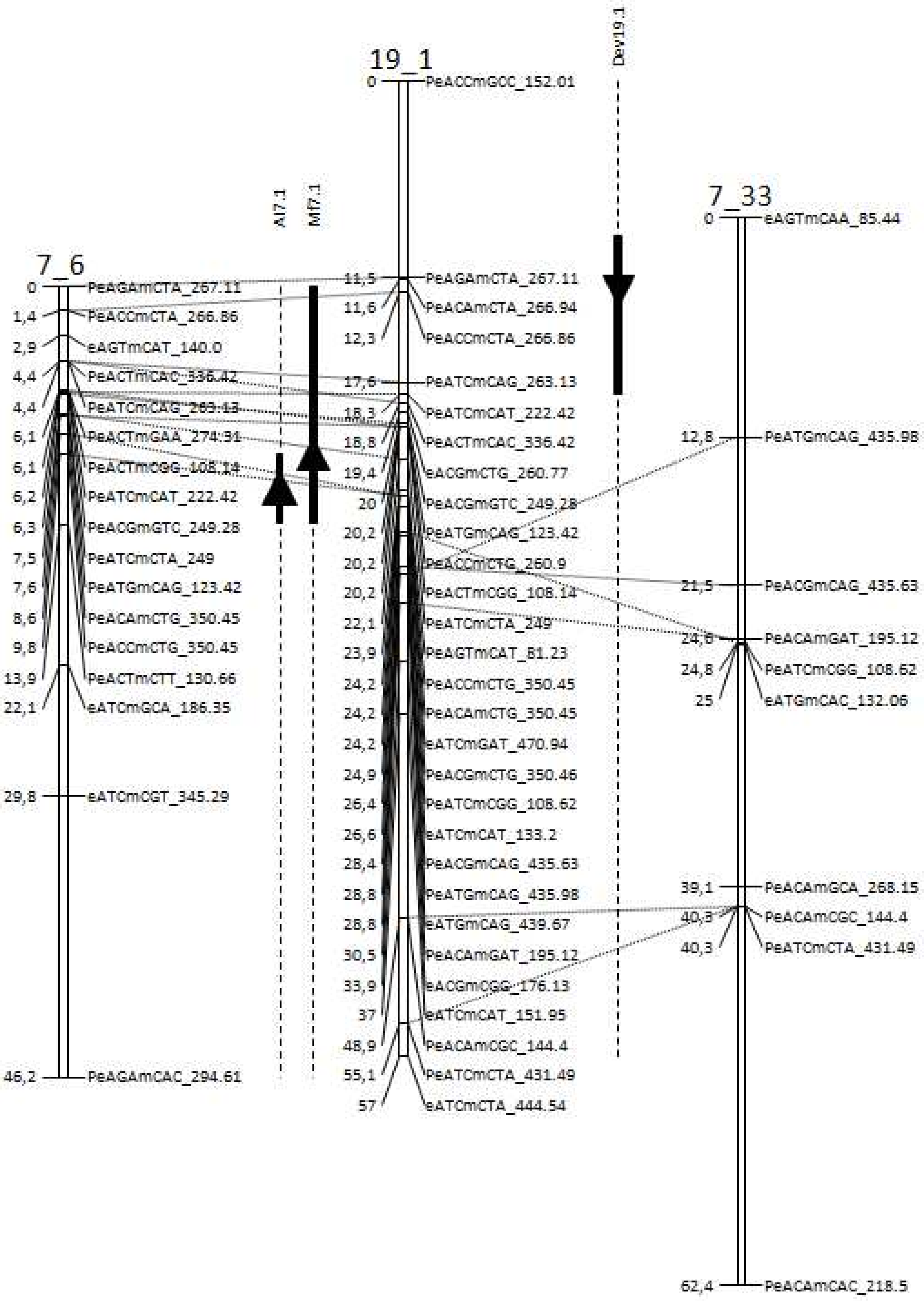

Section 7. Linkage groups 7 and 17 in brood Xt7 and linkage group 6 in brood Xt19; association indicated by common markers.

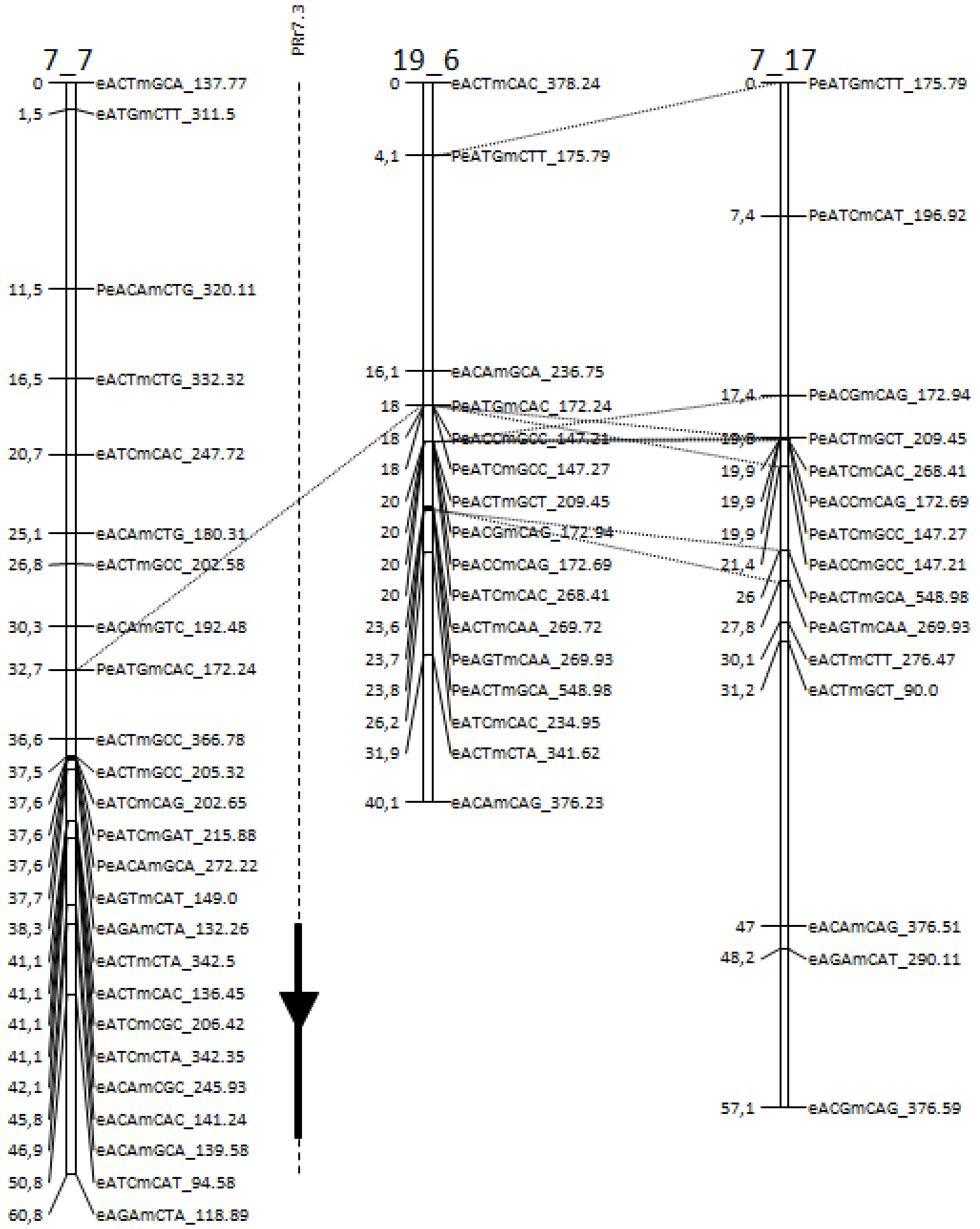

Section 8. Linkage group 10 in brood Xt7 and linkage group 13 in brood Xt19; association indicated by common markers.

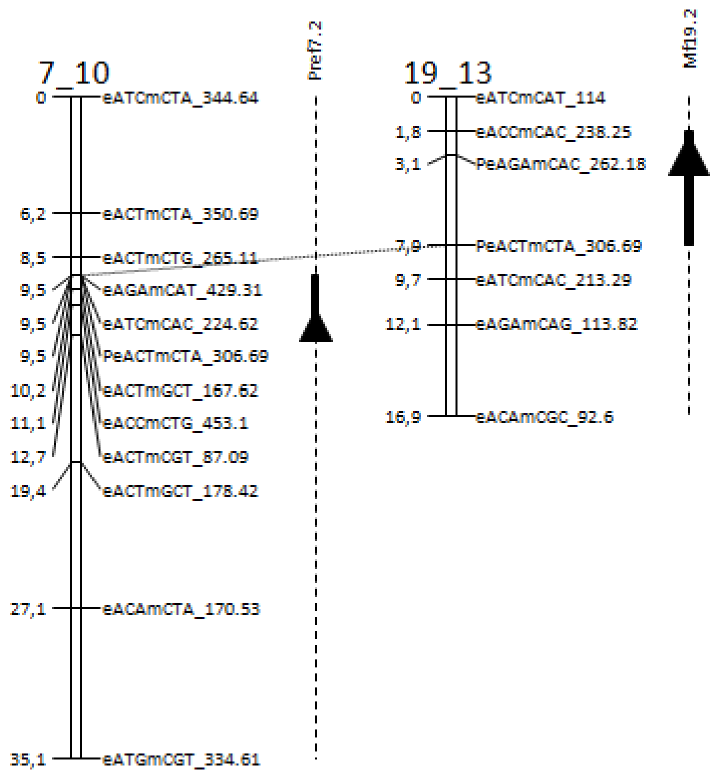

Section 9. Linkage group 11 in brood Xt7 and linkage group 15 in brood Xt19; association indicated by common markers.

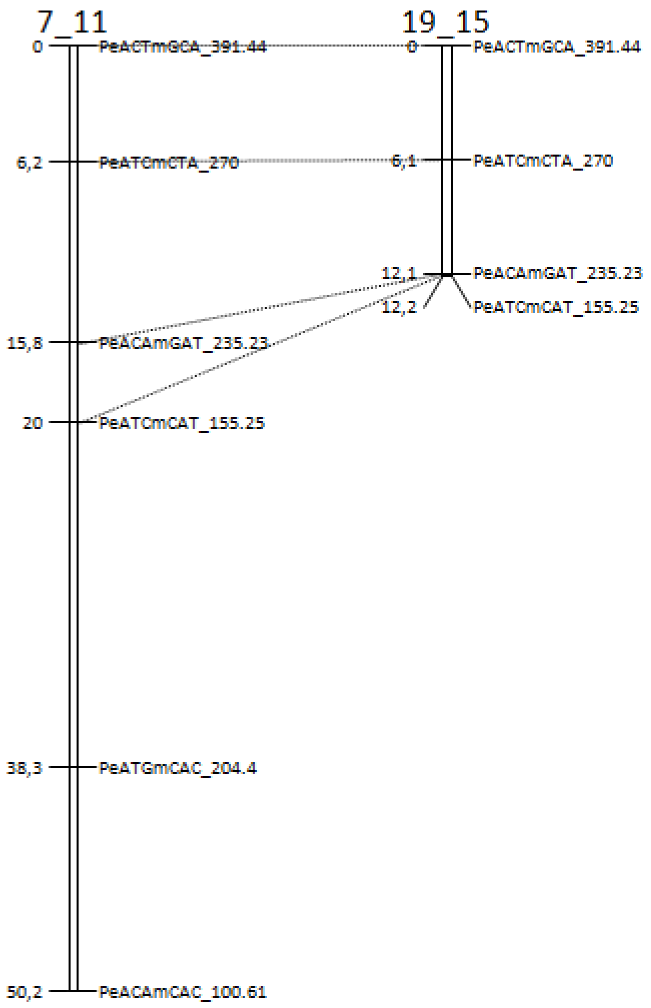

Section 10. Linkage group 12 in brood Xt7 and linkage group 23 in brood Xt19; association indicated by common markers.

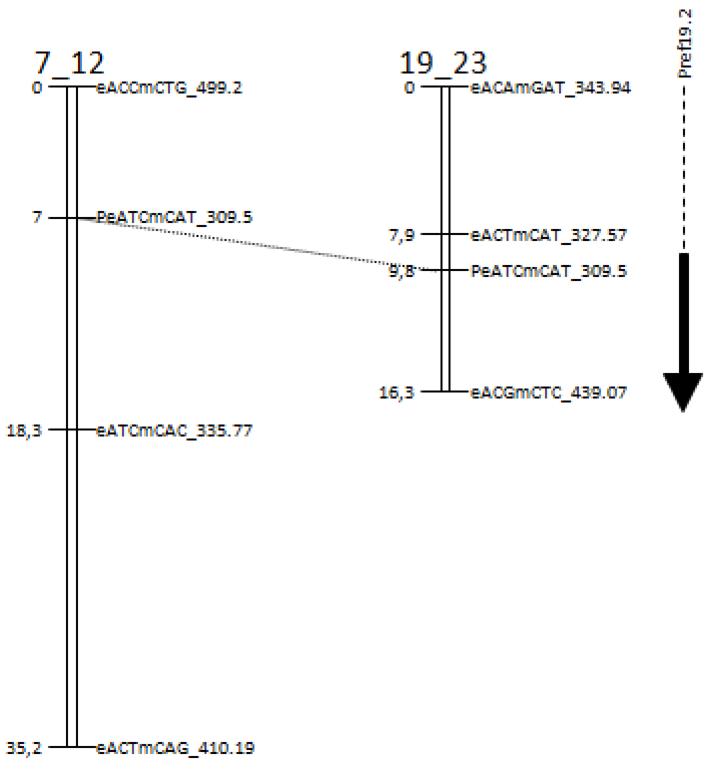

Section 11. Linkage group 13 in brood Xt7 and linkage group 4 in brood Xt19; association indicated by common markers.

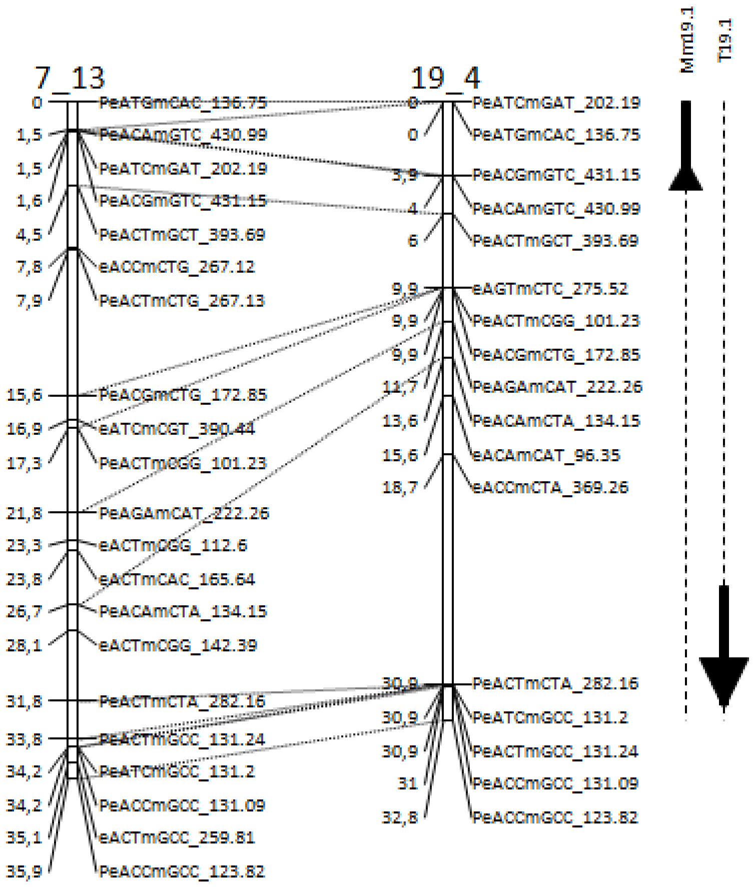

Section 12. Linkage group 14 in brood Xt7 and linkage group 32 in brood Xt19; association indicated by common markers

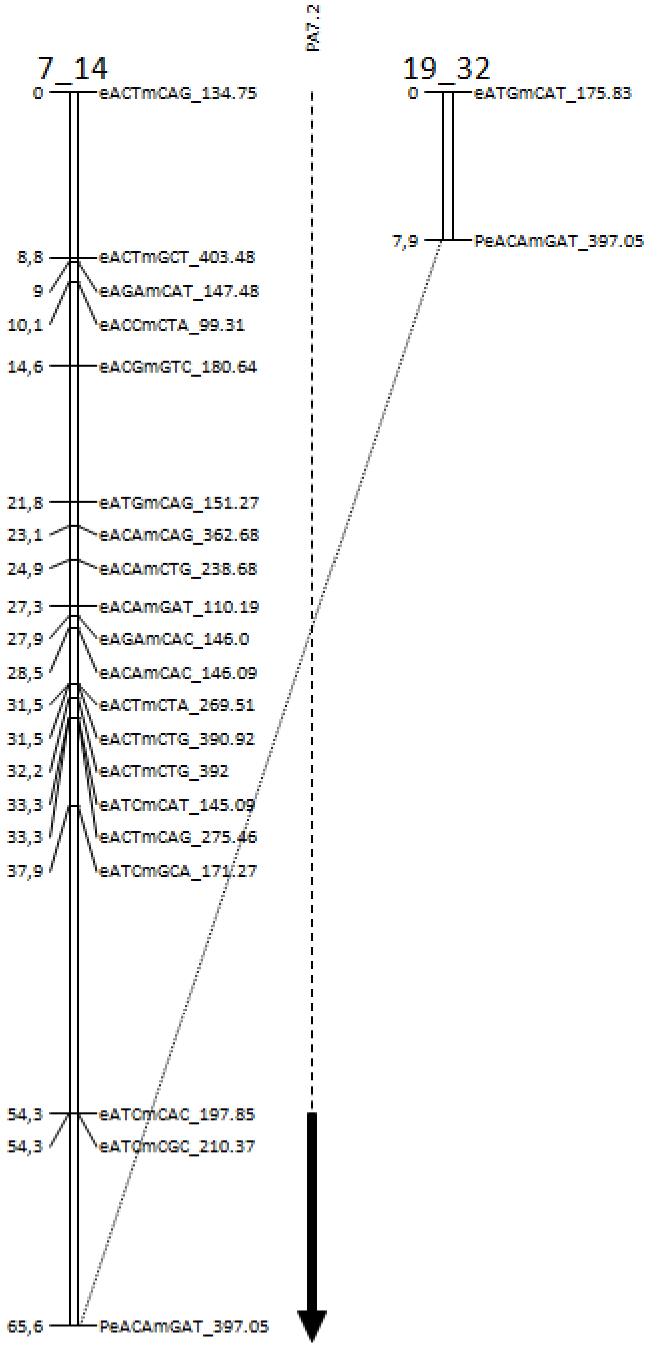

Section 13. Linkage group 16 in brood Xt7 and linkage group 10 in brood Xt19; association indicated by common markers.

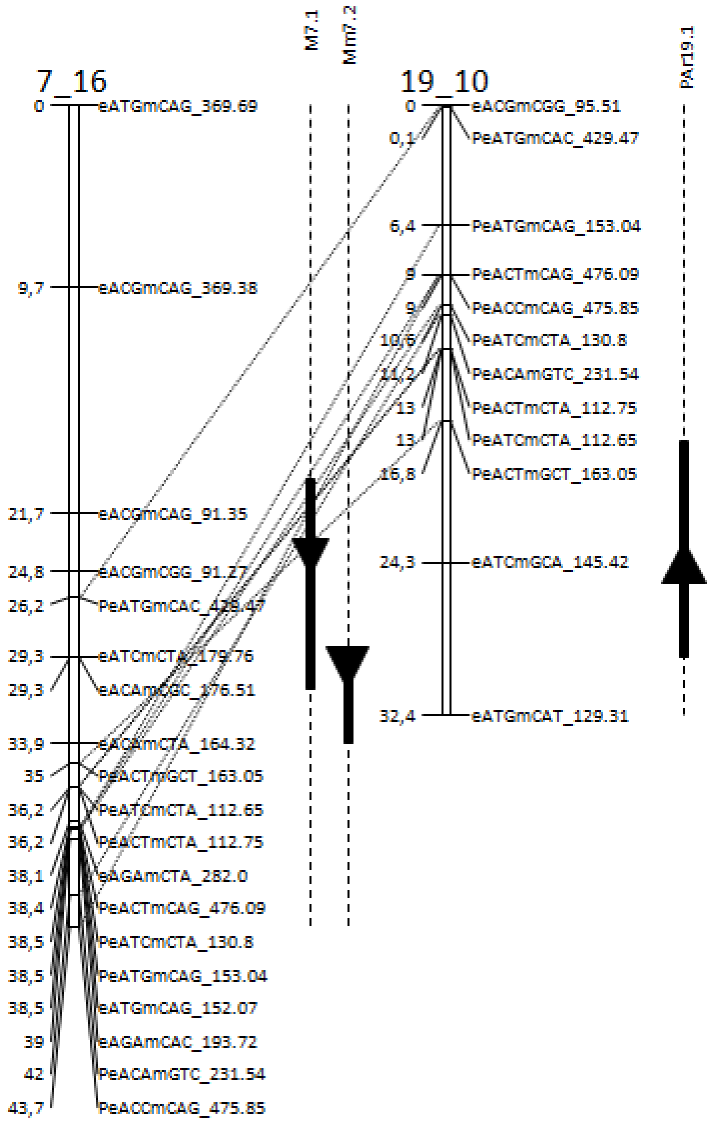

Section 14. Linkage group 18 in brood Xt7 and linkage group 19 in brood Xt19; association indicated by common markers.

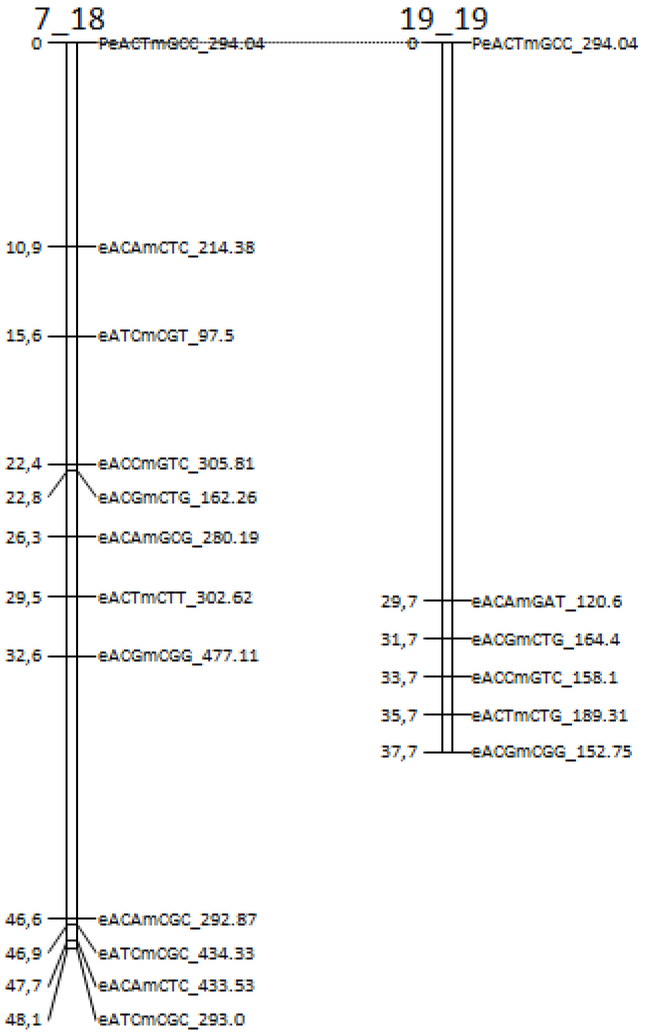

Section 15. Linkage group 21 in brood Xt7 and linkage group 26 in brood Xt19; association indicated by common markers.

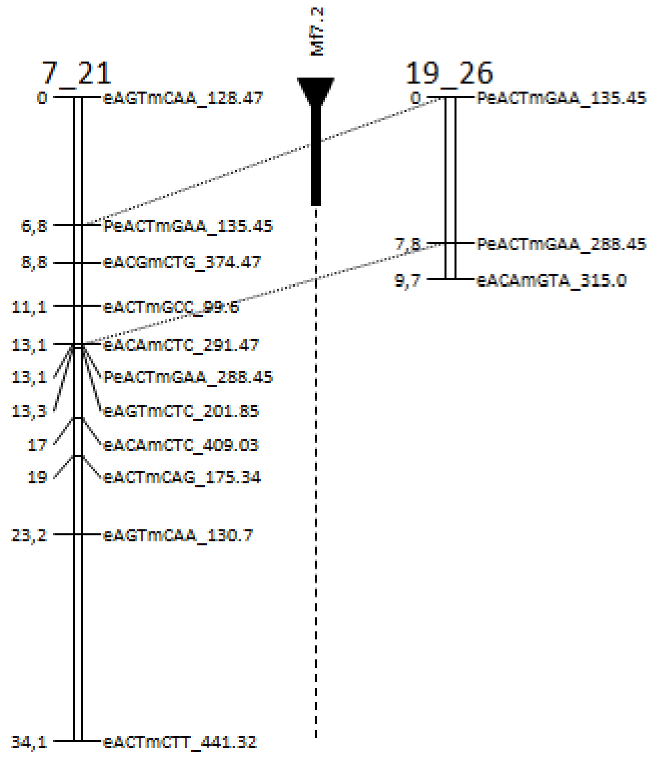

Section 16. Linkage group 22 in brood Xt7 and linkage group 5 in brood Xt19; association indicated by common markers.

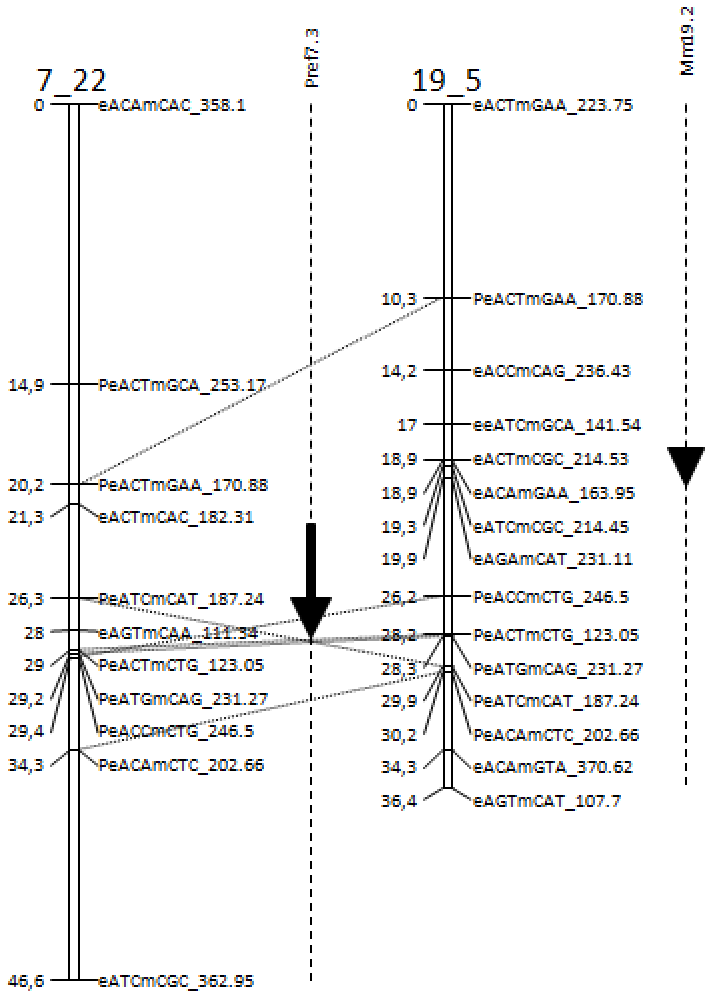

Section 17. Linkage group 24 in brood Xt7 and linkage group 14 in brood Xt19; association indicated by common markers.

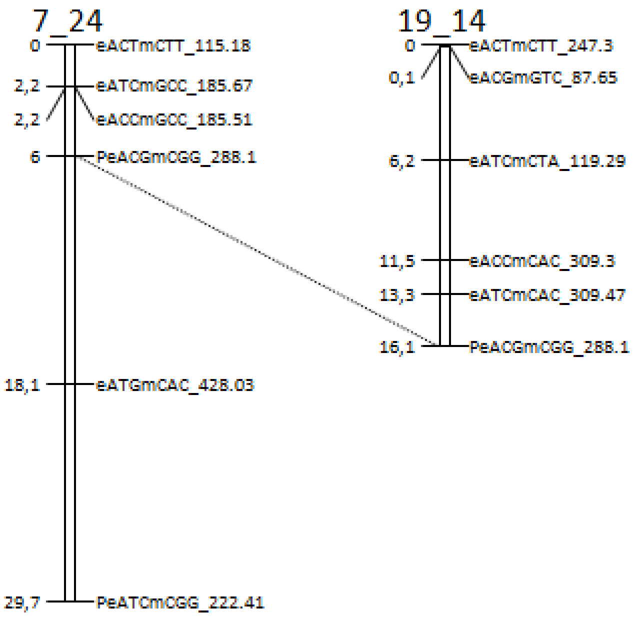

Section 18. Linkage group 26 in brood Xt7 and linkage group 22 in brood Xt19; association indicated by common markers.

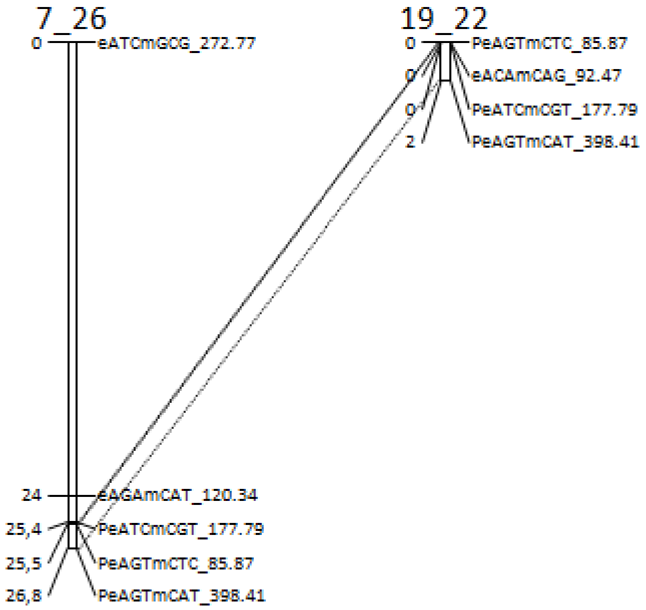

Section 19. Linkage group 27 in brood Xt7 and linkage group 7 in brood Xt19; association indicated by common markers.

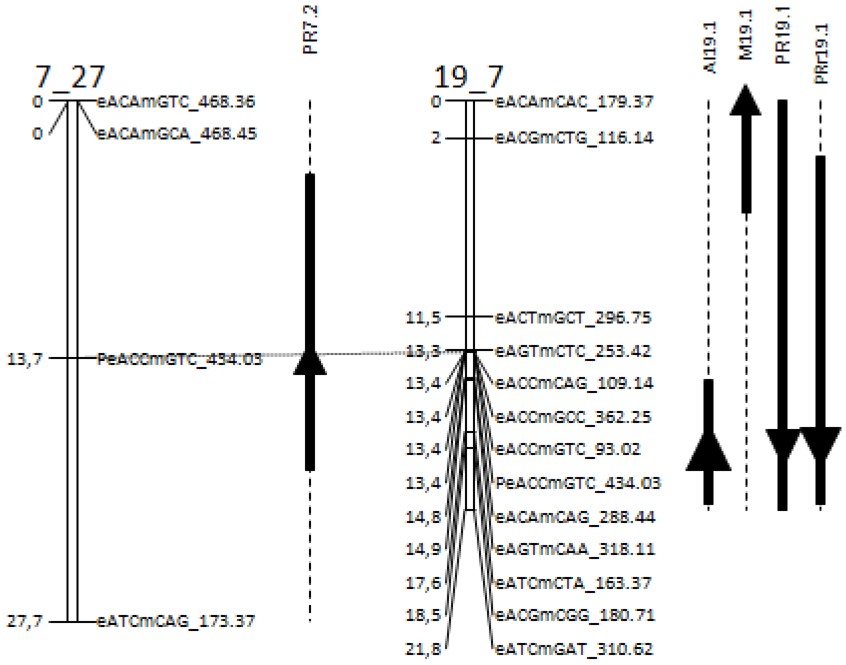

Section 20. Linkage group 28 in brood Xt7 and linkage group 9 in brood Xt19; association indicated by common markers.

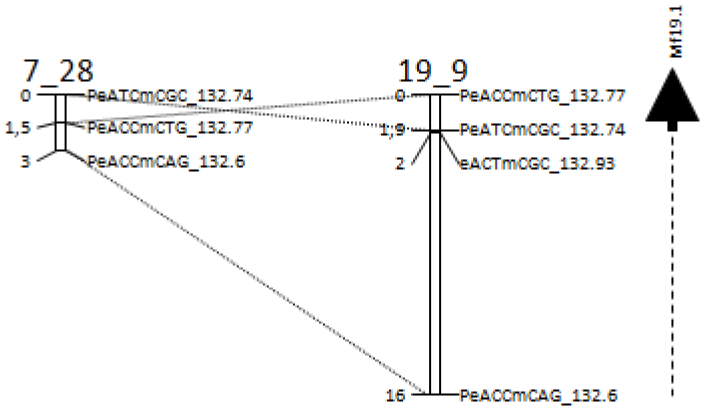

Section 21. Linkage group 29 in brood Xt7 and linkage group 16 in brood Xt19; association indicated by common markers.

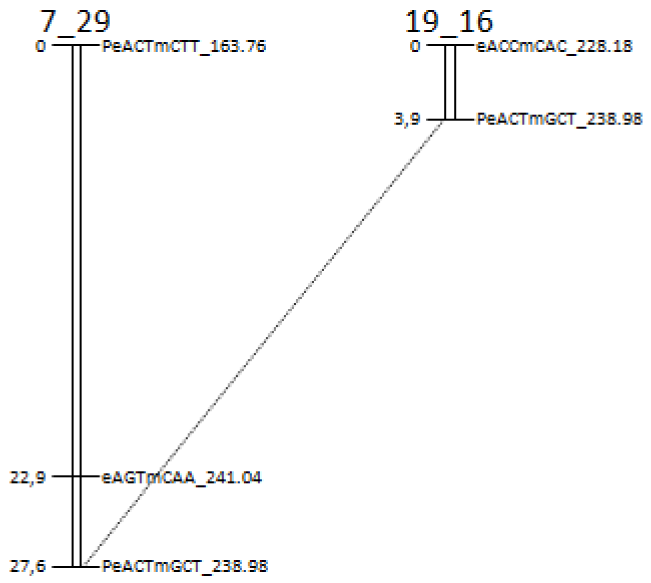

Section 22. Linkage groups 8 and 9 in brood Xt7; absence of common markers prevented association with linkage groups in brood Xt19.

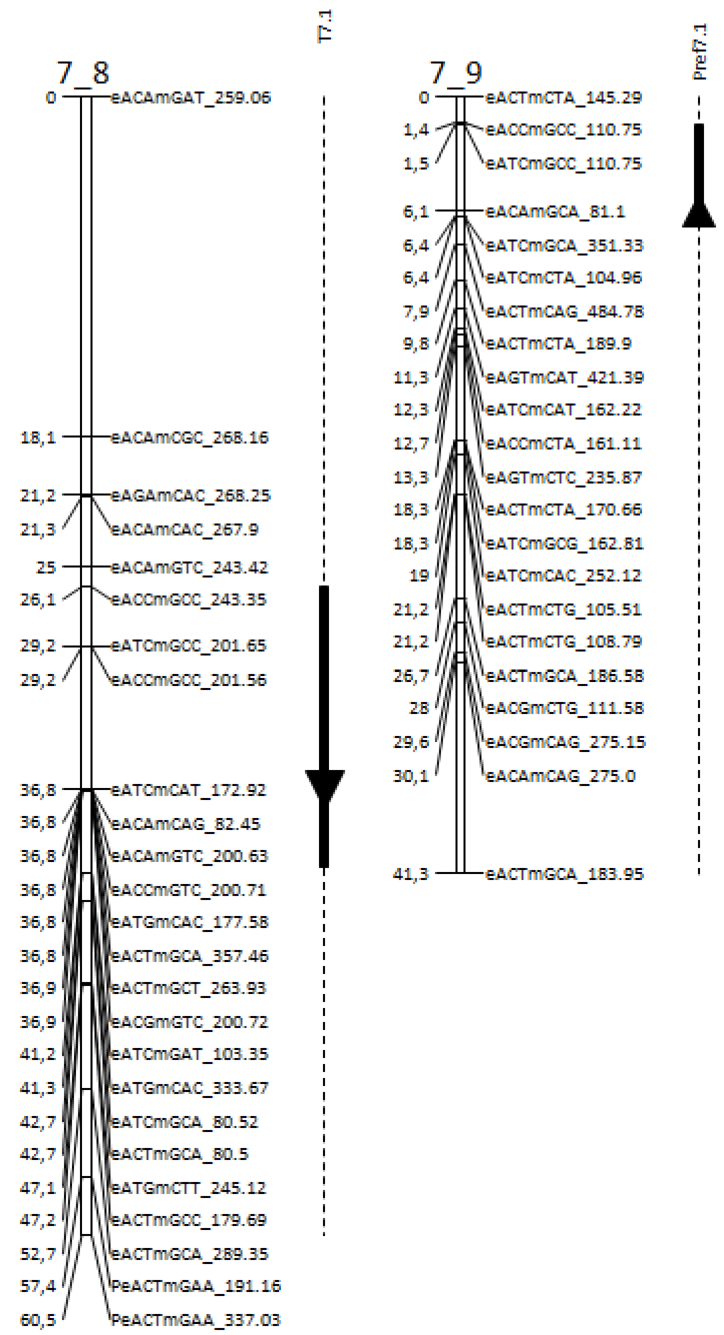

Section 23. Linkage groups 15 and 19 in brood Xt7 ; absence of common markers prevented association with linkage groups in brood Xt19.

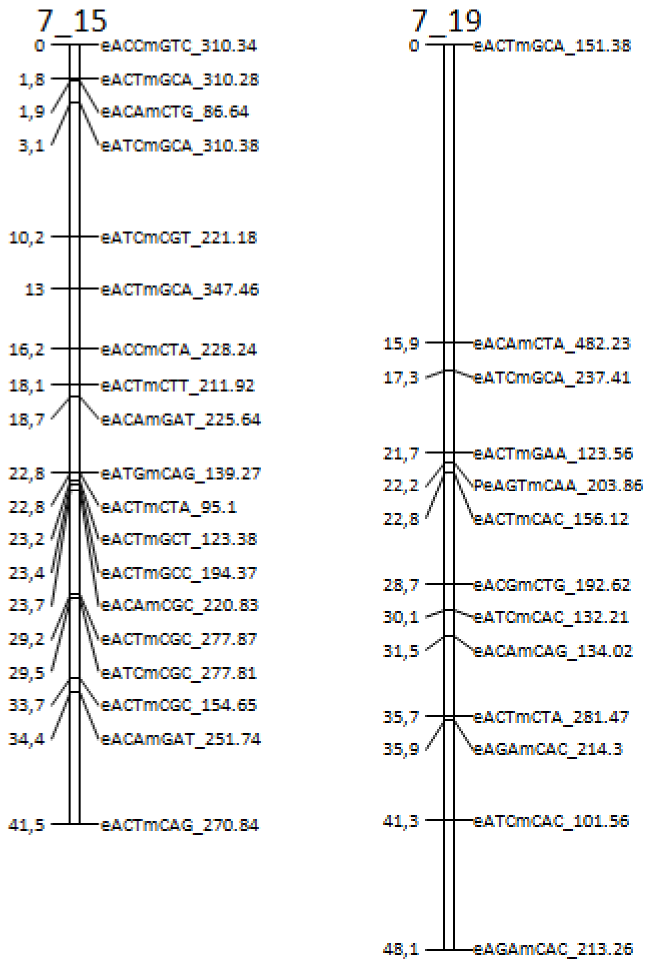

Section 24. Linkage groups 20 and 23 in brood Xt7 ; absence of common markers prevented association with linkage groups in Xt19.

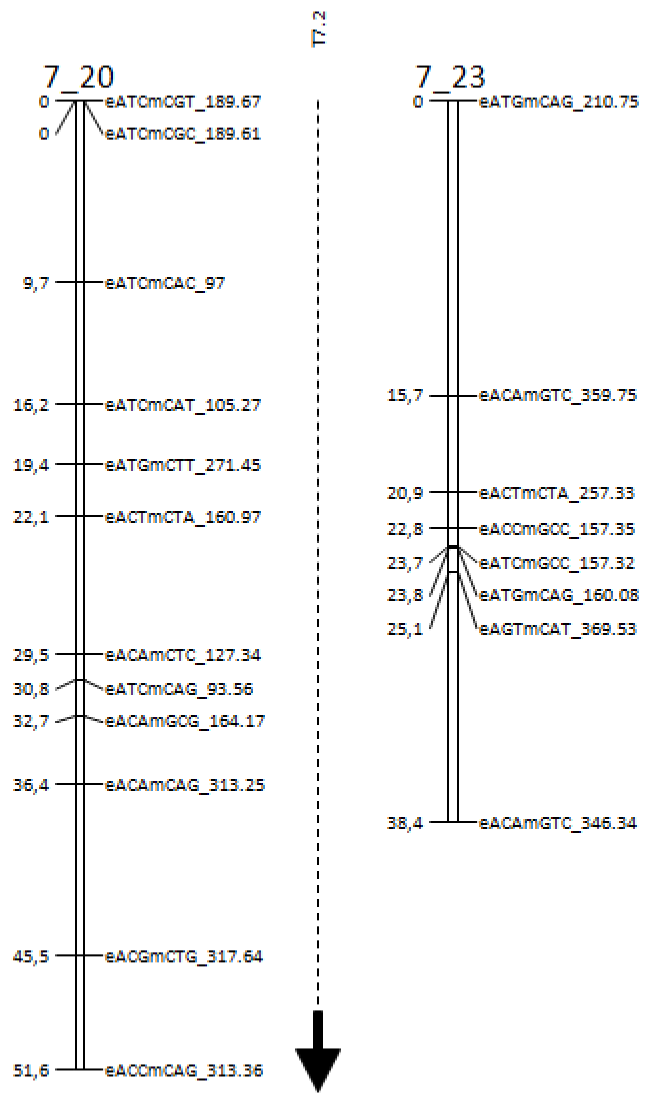

Section 25. Linkage groups 25, 30, 31, and 32 in brood Xt7 ; absence of common markers prevented association with linkage groups in brood Xt19.

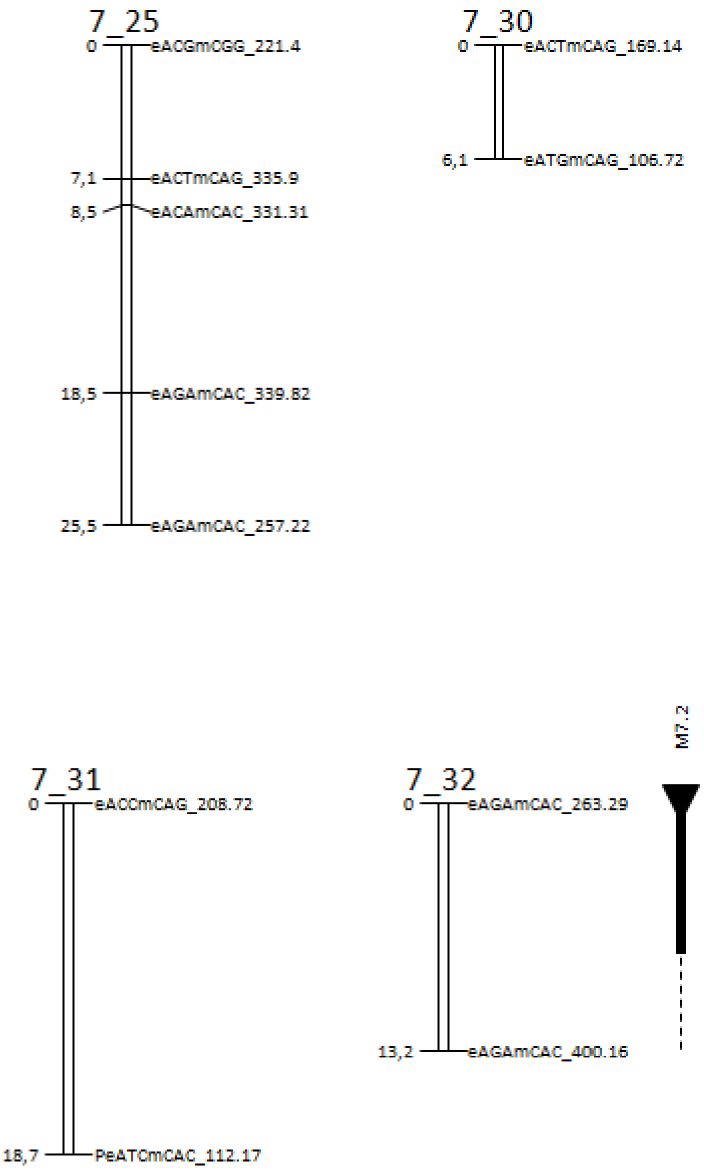

Section 26. Linkage groups 11 and 12 in brood Xt19 ; absence of common markers prevented association with linkage groups in brood Xt7.

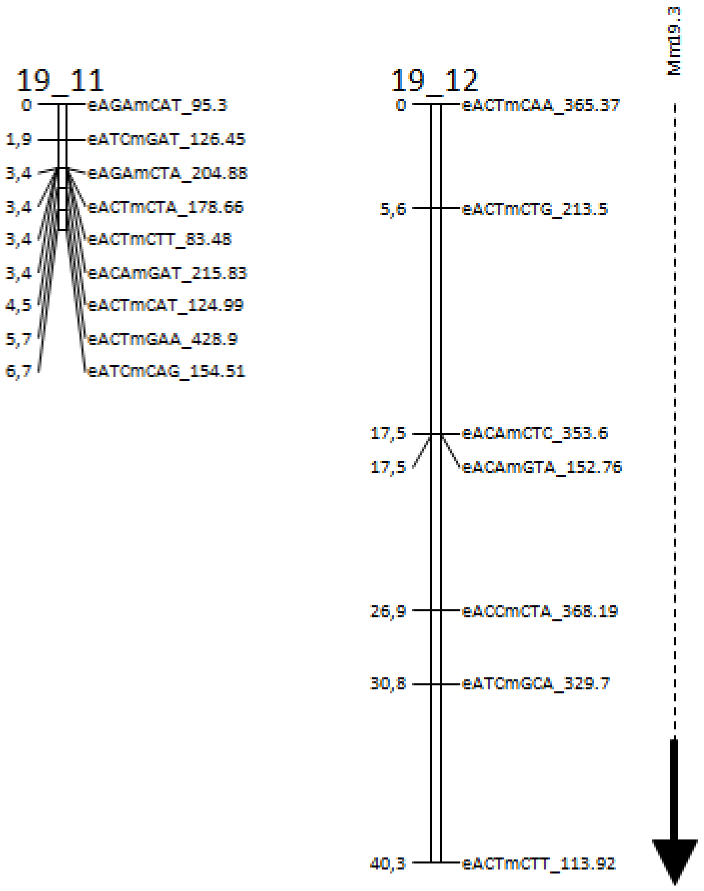

Section 27. Linkage groups 18, 20, 21, and 24 in brood Xt19 ; absence of common markers prevented association with linkage groups in brood Xt7.

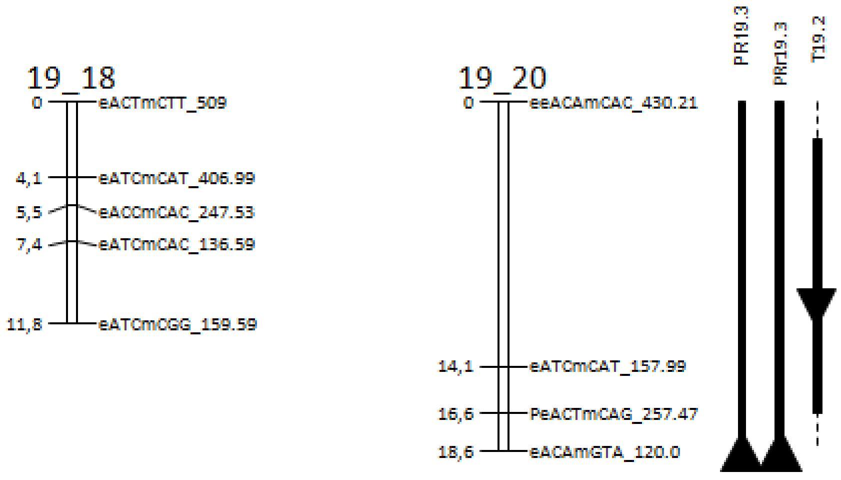

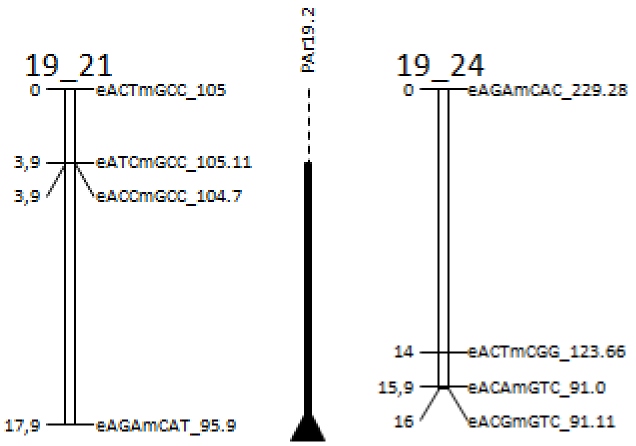

Section 28. Linkage groups 27 and 28 in brood Xt19; absence of common markers prevented association with linkage groups in brood Xt7.

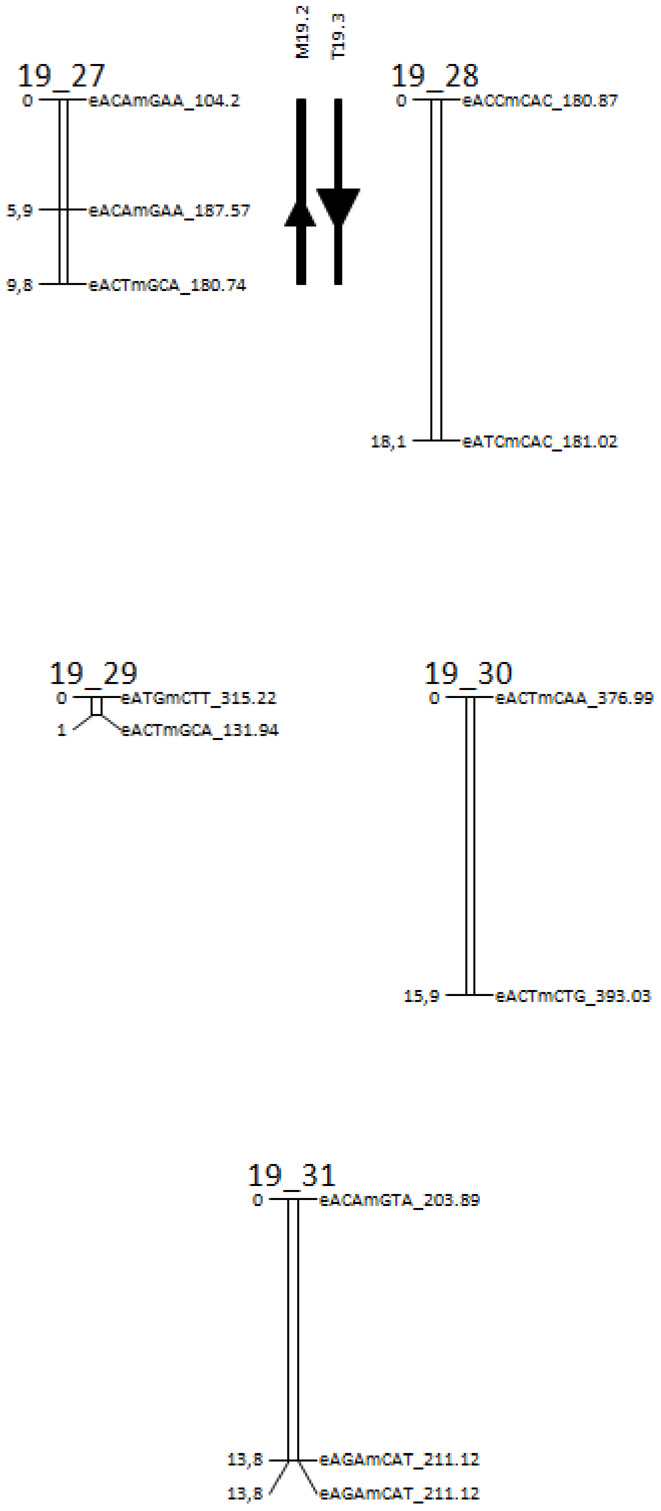

Supplement: Figure S1 — This file illustrates the mapping of QTLs for developmental, male signal, and female receiver traits among linkage groups in broods Xt7 and Xt19. Each section of the file shows maps of a pair of associated linkage groups where association was possible due to common markers, or only one linkage group where common markers did not occur. Two sections (6 and 7) show three linkage groups because linkage group 1 in Xt19 was associated with two linkage groups, 6 and 33, in Xt7, and linkage group 6 in Xt19 was associated with two linkage groups, 7 and 17, in Xt7. For each linkage group map in every section, AFLP markers are listed on the right and their locations, measured in cM (estimated by the Kosambi mapping function) from the telomere, are shown on the left. Lines that connect the maps of associated linkage groups indicate the common markers. Solid triangles to the right of a linkage group map indicate the position of a detected QTL, with triangles pointing upward and downward representing QTLs that exert positive and negative effects, respectively, on the value of a given trait. Triangle size is proportional to the LOD score, the thick vertical line represents the confidence interval (locations on either side of the QTL at which the LOD score decreases by 1 unit relative to the peak) for location, and the QTL name is listed at the top of this line (see Tables 3 and 4 for corresponding information). (PDF) [file pone.0044554.s001.pdf]
